# Nano-bioremediation of metal-polluted industrial wastewater using myco-synthesized iron oxide nanoparticles derived from *Aspergillus niger* AUMC 16028

**DOI:** 10.1038/s41598-025-19925-4

**Published:** 2025-10-07

**Authors:** Abd El-Raheem Ramadan El-Shanshoury, Metwally Abd El-Azeem Metwally, Nanis Gamal Allam, Hany Eltohamy Hemdan, Wafaa Kamel Abdella, Youssef Moustafa Mohammed

**Affiliations:** 1 Microbiology Section, Botany and Microbiology Department, Faculty of Science, Tanta University, Tanta, 31527 Egypt; 2 Microbiology Department, Holding Company for Water and Wastewater, Cairo, Egypt; 3 Botany and Microbiology Department, Faculty of Science, Damanhour University, Damanhour, Egypt

**Keywords:** Biotechnology, Microbiology, Environmental sciences

## Abstract

The presence of heavy metals in wastewater poses serious ecological and environmental issues. Using biogenic nano adsorbents to remove heavy metals from industrial wastewater could be beneficial and serve as an alternative to traditional chemical and physical methods in real-world applications. The aim of this study is to biosynthesize green iron oxide nanoparticles (IONPs) for the removal of heavy metals from industrial wastewater. This process utilizes a cell-free extract derived from heavy metal-resistant fungi that were isolated from various industrial wastewater effluents in Egypt. Several fungal strains were examined for their ability to produce IONPs. A molecular identification of the most powerful fungus was made. The color change, as observed using UV-Vis spectroscopy, indicated that IONPs were being produced. Box-Behnken design (BBD) and Plackett-Burman design (PBD) were used to optimize the mycosynthesis of IONPs. The iron oxide nanoparticles (IONPs) produced through mycosynthesis were characterized using several techniques, including Fourier-transform infrared spectroscopy (FT-IR), X-ray diffraction (XRD), energy dispersive X-ray spectroscopy (EDAX), scanning electron microscopy (SEM), and transmission electron microscopy (TEM). After characterization, we evaluated their ability to extract heavy metal nanoparticles from both industrial and synthetic wastewater effluents. The results showed that different levels of IONPs were formed by various fungal strains: *Aspergillus niger* strain F1, *A. flavus* strain F2, *Mucor sp.* strain F3, and *Alternaria sp.* strain F4. Molecular analysis identified the most effective fungus for IONP production as *A. niger* AUMC 16028. The myco-synthesized IONPs were validated through the analyses conducted. Optimal conditions for IONP myco-synthesis included 8 g/L of yeast extract, a reaction temperature of 40 °C, and a culture period of 6 days. The myco-synthesized IONPs achieved heavy metal removal efficiencies of 92.47% for copper (Cu²⁺), 72.77% for iron (Fe³⁺), 84.76% for manganese (Mn²⁺), 70.28% for zinc (Zn²⁺), and 80.79% for chromium (Cr³⁺) in synthetic wastewater. Furthermore, the removal efficiencies of Zn²⁺ and Fe³⁺ in industrial effluent were 78.75% and 90.74%, respectively. These findings demonstrate that the heavy metals copper, iron, manganese, zinc, and chromium were effectively removed from synthetic wastewater, as well as iron and zinc from industrial wastewater, through the myco-synthesis and optimization of IONPs derived from *A. niger* AUMC 16028. This research offers a promising, green environmentally friendly, and efficient method for long-term industrial wastewater bioremediation and contributing to additional clean water resources.

## Introduction

Water pollution is becoming a principal global concern because of rapid industrialization, inefficient farming practices, and an increase in human population and activity. Heavy metals and organic and inorganic pollution are all major contaminants^1^. Numerous researchers have focused on removing hazardous released metals because they can cause serious illnesses in plants, animals, and environmental components^2^. In this regard, Gupta and Shukla^3^ proposed that advanced oxidation methods and valorization strategies can reduce the toxicity of wastewater effluents and promote sustainable use. The production of biogenic nanoparticles (NPs) has been shown to be a successful research field.

Biogenic NPs must be produced using live organisms. These organisms are simple to grow and have a high intracellular metal absorption capacity. Because of their metabolic processes, these organisms can synthesize NPs either extracellularly or intracellularly via a different synthesis pathway. Biogenic processes allow for the use of natural products instead of hazardous chemicals, offering an environmentally and economically viable solution. Researchers are investigating the manufacturing of NPs from microbial resources, often referred to as bio-factories^4–6^. These microorganisms can create metal NPs both inside and outside cells, making them a potential area in nanobiotechnology. Nanobiotechnology is becoming more widely used due to its numerous benefits. Eco-friendly and sustainable nanoparticle synthesis is becoming more popular due to concerns about the long-term risks of traditional chemical methods^7,8^. The process involves microbial isolation, growth, and maintenance, that is considered safe, easy, cheap, sustainable, and environmentally friendly, aligning with “green chemistry” goals^9,10^.

Bacteria possess characteristics related to biomineralization, bioaccumulation, and bioleaching, which can transform metals. This has led to interest in using bacteria to create nanoparticles^11–15^. Fungal cultures generate more biomass and are used as reducing and stabilizing agents, despite their resistance to heavy metals and ability to absorb and bioaccumulate metals. Furthermore, several fungal species may easily be maintained on huge scales in “nano factories,” generating a range of metal NPs with regulated size and form^16^.

Mycoremediation refers to fungus-based remediation; fungi are naturally good scavengers with a high metal uptake capacity because they contain enzymatic machinery found both inside and outside of cells that removes heavy metal ions through breakdown and biosorption. As a result, fungi served as a possible bioremediation agent. Heavy metal ions were removed from polluted water by a number of filamentous fungi, especially *Aspergillus flavus*, *Penicillium*, *Trichoderma*, *A. niger*, and *A. fumigatus*^17,18^. Filamentous fungi are favored by nanotechnologists due to their advantages over bacteria, yeast, actinomycetes, and plants because of their simplicity of handling, minimal dietary needs, strong wall-binding capacity, and ability to absorb minerals into cells^19^.

Biogenic NPs have shown significant effectiveness in eliminating contaminants like heavy metals, organic molecules, inorganic compounds, and pharmaceutical residues from wastewater^13,20^. These nanomaterials must be nontoxic, have high adsorption capabilities, be selective for low pollutant concentrations, be easy to remove, and be highly recyclable. Heavy metals have been extracted from wastewater using a variety of nanomaterials, like carbonaceous, chitosan, magnetic, zeolite, bimetallic, metallic polymer-based ferrite, and metal oxide^21^. Hybrid nanostructures, metals, and metal oxides were used to increase catalytic efficiency and chemo-adsorption^22,23^.

Iron-based NPs, a potential new resource for water treatment because of their large surface area, functionalization, low cost, magnetic properties, and antimicrobial and antioxidant activities, are also used in numerous fields such as wastewater treatment^24^paint, textiles, food, medicine, and cosmetics, making them a significant synthetic material^25^. IONPs are the smallest iron metal particles, with great thermal and electrical conductivity, huge surface area, outstanding dimensional stability, magnetic properties, non-toxicity, and high reactivity. IONPs can rapidly oxidize and liberate free iron ions if exposed to air or water, and they have a variety of applications^26^. Magnetic nano adsorbents are made from iron oxide nanoparticles, which have a strong magnetic attraction and superparamagnetic reactivity. Recycling these nanoparticles is easier and more convenient because they lose magnetism when placed under an external magnetic field. Because of these properties, iron oxides make excellent nanocatalysts for removing heavy metals from pollution^27^. Fungal species are recognized for their ability to produce IONPs, because of the great potential of using phytochemicals extracted from them as a very suitable choice for the biofabrication of Fe_3_O_4_ nanoparticles, perfect for immobilizing metals and producing NPs because of their resistance to metal toxicity which may find applications in biomedicine and bioremediation^28–31^.

Fungi’s resistance to metal toxicity and the phytochemicals that can be derived from them make fungi ideal for immobilizing metals and creating NPs. Fungi synthesize metallic nanoparticles through complicated metabolic processes, utilizing their unique properties. They use their metabolic pathways to convert metal ions into nanoparticles, which they can manufacture either inside or outside the cell. Compared to bacterial systems, fungal cultures produce a higher amount of biomass and don’t need complicated extraction procedures recover the filtrate and biosynthesize the nanomaterials ^28,32–34^.

Although the technique of microbial nanoparticle production is still not limited, microorganisms help to synthesize stable nanoparticles with good properties. Biogenic nanoparticles have a high surface area, which enhances their adsorption capacity. Size and form can be influenced by pH differences, substrate availability, and reaction contact time. Over time, many remediation strategies have been developed to treat heavy metal contamination produced by both natural and industrial activity. Biogenic techniques allow for the use of natural ingredients rather than hazardous chemicals, making it an environmentally and economically acceptable solution. The integration of nanotechnology with bioremediation may prove to be an appropriate approach and a safer alternative to conventional techniques. Thus, adopting a greener method may result in a significant reduction in heavy metal contamination and hazardous consequences, as well as a reduction in overall cost and cleanup time. Fungi are well-known for producing a diverse spectrum of bioactive chemicals and transforming metal ions into nanoscale forms. Fungi-produced iron oxide nanoparticles have a large surface area and reactivity, allowing them to effectively absorb and/or convert metal contaminants, cleaning and recycling wastewater. Extensive research on several nanoparticle-related topics is required prior to their commercialization. Future research should focus on bridging the gap between lab-scale synthesis and industrial-scale output. As a result, in this study, a few fungi resistant to iron ions were selectively isolated from industrial wastewater effluents, followed by the selection of the most successful fungal strain (*Aspergillus niger* AUMC 16028) for producing iron oxide nanoparticles (IONPs). Furthermore, the green-produced IONPs have been characterized and tested as a heavy metal adsorbent and removal from synthetic and real industry wastewater to safeguard the environment from pollution and introduce non-traditional water resources.

## Materials

All chemicals and reagents used in this study were analytical grade. Ferric chloride (FeCl₃·6 H₂O, ≥ 99% purity), copper sulfate (CuSO₄·5 H₂O, ≥ 98% purity), manganese sulfate (MnSO₄·H₂O, ≥ 98% purity), zinc sulfate (ZnSO₄·7 H₂O, ≥ 98% purity), and chromium (III) chloride (CrCl₃·6 H₂O, ≥ 98% purity) were purchased from Sigma-Aldrich (USA). Distilled water was used throughout all experiments. A 0.1 M HCl solution and aqueous NaOH were used to adjust the pH.

## Methods

### Collection of water samples contaminated with metals

Four industrial wastewater samples were collected from El-Gharbia Governorate, Egypt. They were collected from the effluent of Kaha Chemicals and Operations Company, Cairo Petroleum Refining Company, El-Etihad and Al-Tabrid Company, and Drinking Water and Sanitation Company. The industrial wastewater samples were collected in sterilized sampling containers and transferred to the lab under standard aseptic conditions. The samples were preserved at a temperature below 4 °C until needed^35^.

### Isolation of fungi from metal-polluted wastewater

One mL of industrial effluent had been added to a 100 mL Erlenmeyer flask containing 10 mL of sterilized distilled water and rapidly shaken. Serial dilutions were done; one mL of the appropriate dilution was cultivated on potato dextrose agar (PDA) medium plates that were incubated for 7 days at 28 °C. The various fungal colonies that developed on the inoculation plates were chosen and transferred to new plates for purification. The purified fungi were put on slant culture tubes of PDA medium, kept at 4 °C, and subcultured every 6 months^36^.

### Screening for extracellular production of iron oxide nanoparticles

One milliliter of spore suspension (10^6^ spores/mL) of selected fungal isolates was inoculated separately in 250-milliliter conical flasks containing 100 milliliters of potato dextrose broth (PDB) medium at pH 5.0 and incubated at 28 °C for 7 days. Following incubation, the growing fungal biomass was filtered through filter paper in a biosafety cabinet, and any media components were eliminated by washing it with sterilized deionized water.

Twenty grams of the fungal biomass that had been collected were transferred to 250 mL Erlenmeyer flasks with 100 mL of sterilized distilled water. Flasks were incubated at 28 °C in a rotary shaker (150 rpm) for 72 h. Following incubation, the cell-free filtrate was obtained by filtering it through filter paper^37^. An equal volume of 1 mM FeCl₃·6 H₂O solution was mixed with each isolate’s supernatants individually and incubated for 48 h at 28 °C and 150 rpm in the dark on a rotary shaker, with continuous checks. In addition to the experimental flasks, a control flask (free of iron ions) was used^38^. Next, most of the potent fungal isolates used in the biosynthesis of IONPs were chosen based on the color change and maximum absorption resonance peaks determined using UV-visible spectroscopy (JENWAY 6305). To obtain the NPs, the fungal mycelium was filtered using filter paper. The filtrate was centrifuged for ten minutes at 10,000 rpm then washed with deionized water. The purified pellets were then placed in petri dishes and dried for 24 h in a 60 °C oven. The resulting powdered nanoparticles that were created were used for further studies.

### Identification of the most effective isolate for producing iron oxide nanoparticles

Conventional techniques were used to identify the most efficient fungi that generate IONPs based on macro- and micromorphological characteristics^39,40^. The chosen isolate’s hyphal morphology was examined using a light microscope (LM) after being stained with lactophenol cotton blue. The chosen isolate was then sent to the Molecular Biology Research Unit, Assiut University, for extraction of DNA utilizing a Patho-gene-spin DNA/RNA extraction kit donated by Intron Biotechnology Company in Korea. The fungal DNA was sent to SolGent Company in South Korea for sequencing of the internal transcribed space (ITS) region of the fungal rDNA and polymerase chain reaction (PCR). The ITS region was amplified utilizing the universal primers ITS1 Forward (5’-TCCGTAGGTGAACCTGCGG-3’) and ITS4 Reverse (5’-TCCTCCGCTTATTGATATGC-3’) using an ABI 9700 thermal cycler. Purified PCR products were sequenced utilizing the same primers with the incorporation of dideoxy-nucleotides (ddNTPs) in the reaction mixture. The acquired sequences were analyzed utilizing the Basic Local Alignment Search Tool (BLAST) from the National Center for Biotechnology Information (NCBI) website. The resulting sequence, as well as those received from the GenBank database, were submitted to Clustal W analysis utilizing MegAlign software version 5.05 (DNASTAR Inc., Madison, Wisconsin, USA) for phylogenetic analysis. Furthermore, the sequences were forwarded to GenBank to get the accession number^41^.

### Characterization of iron oxide nanoparticles

#### UV–Visible spectroscopic analysis

The absorbance was scanned in the range of 200–800 nm. A UV-Vis spectrophotometer was used on a regular basis to measure the bio-reduction of soluble salts to NPs after the samples were diluted with deionized water. A UV-Vis spectrophotometer of IONPs was made with a quartz cuvette and water as a reference^42^.

#### Scanning electron microscope analysis

Nanoparticles’ average shapes were assessed by scanning electron microscopy. Following sonication, a drop of IONPs solution was applied to a glass slide and allowed dry before being suspended in double distilled water. Platinum plating was used to turn them into conductors^43^.

#### Fourier-transform infrared spectroscopic analysis

Fourier-transform infrared spectroscopic (FTIR) spectra were used to study the functional groups on IONPs surfaces. Utilizing a Bruker alpha spectrophotometer (Perkin Elmer, USA) with a 4 cm⁻¹ resolution, the FTIR analysis was carried out. A disk with a sample of 2% dried IONPs and 50 mg KBr was made. The sample was scanned at 400–4000 cm^− 1^^44^. The reference chart and the spectral data were compared to identify the functional groups found in the sample.

#### X-ray diffraction spectroscopy analysis

X-ray diffraction spectroscopy (XRD) analysis of NPs was determined using CuKα1 radiation (1.5406 Å; 35 kV, 25 mA). The XRD analysis was examined to establish location, width, and peak intensity. Using the Debye-Scherrer equation, the size of the NPs was determined.

1 $$\:D=K\lambda\:/\beta\:cos\theta\:$$

Where D represents the size of IONPs, Scherrer’s constant is k (0.9), λ represents the wavelength of the X-ray radiation source (λ = 1.5406 Å), the Bragg’s diffraction angle is θ, and β represents the width of the XRD spectrum at semi-height^45^.

#### Transmission electron microscopy analysis

Transmission electron microscopy (TEM) (JEOL JEM-2100) with an accelerating voltage of 200 kV was used to analyze the dimensions and shape of the myco-synthesized IONPs. Samples for TEM examination were generated by dropping IONPs solution onto carbon-coated copper grids. Samples were dried and stored under vacuum in desiccators before being loaded onto a specimen holder. Minitab statistical software (X64/21.3.1.0) and ImageJ were used to calculate the diameter of NPs and the average size variation from TEM micrographs^46^.

#### Energy dispersive x-ray analysis

The structure of IONPs was determined using an energy dispersive x-ray (EDAX) spectrum via an X-ray micro-analyzer (Module Oxford 6587INCA X-sight) connected to a (JEOL JSM 5500 LV) SEM to confirm the existence of iron in the particles and to determine their other elementary compositions. Elemental analysis was conducted at 20 keV^47^.

### Optimization studies on myco-synthesis of iron oxide nanoparticles

The Plackett-Burman fractional factorial design (PBD) and the Box-Behnken design (BBD) were used to test the influence of different variables on the myco-synthesis of IONPs from chosen isolates.

#### Plackett-Burman design

The Plackett-Burman fractional factorial design (PBD) was utilized to screen process parameters, which is an effective method for selecting significant factors from many variables that might impact the process. (PBD)^48,49^ was utilized to investigate the various factors affecting the myco-synthesis of IONPs from chosen isolates. In twelve trials, eleven independent variables were screened using Minitab 16 software and the (PBD) matrix. Eleven independent variables were chosen: culture age, culture temperature, culture pH, yeast extract, inoculum size, biomass contact time, reaction time, reaction temperature, reaction pH, Fe^3+^, and reaction shaking. Each variable was examined at both a low (-) level and a high level (+) (Table 1). All experiments were done in triplicate. The myco-synthesis of IONPs was tested by measuring the absorbance of the resultant mixtures at 410 nm with UV-visible absorption spectroscopy, as well as the iron removal efficiency. Equation (2) was used to calculate the effect of each individual variable.

2 $$\:{E}_{xi}=(\sum\:{M}_{{i}^{+}}-\sum\:{M}_{{i}^{\_}}\:)\backslash\:N$$

Where E_xi_ was the influence of the tested factors, Mi + and Mi − represent the absorbance of myco-synthesis of IONPs with high and low levels, respectively, and N was the number of trials divided by 2. A positive main effect figure indicates that the factor’s highest value is near to the optimum, while a negative sign indicates the factor’s minimal value is near to the optimum. The myco-IONPs-based factorial experiment was investigated by ANOVA and regression. The regression analysis revealed that the significant factors had a higher effect on Myco-IONPs^50^. The data analysis (p-value, confidence level, and t-value) was carried out utilizing Microsoft Excel to assess the significance of the variables^51^.

**Table 1:** Factors and levels in Plackett-Burman statistical design affecting myco-synthesis of iron oxide nanoparticles.

Variables	Unit	Level	
		Low (−)	High (+)
Culture age	(days)	5	9
Culture temperature	⁰C	25	35 °C
Culture pH		5	8
Yeast extract	g/L	0	5
Inoculum size	disk	1	2
Biomass contact time	day	1	3
Reaction time	h	24	48
Reaction temperature	⁰C	25	35
Reaction pH		5	8
Fe^3+^	mM	1	3
Reaction shaking	rpm	0	150

#### Box-Behnken design

The Box-Behnken statistical design (BBD)^52^ was employed via Minitab 16 software. Based on PBD results, we designed BBD to optimize the three factors (yeast extract, reaction temperature, and culture age). Three independent variables were tested in fifteen trials and organized using the BBD. Each factor was assigned to three levels (-1, 0, and 1) and labeled (Table 2). All trials were conducted in duplicate, and the experimental data was fitted using polynomial regression analysis, resulting in a quadratic polynomial. According to^53^, the resulting model can explain the relation among the influencing and response factors using Eq. (3):

3 $$\:\varvec{Y}=\:{\varvec{\beta\:}}_{0}+{\sum\:}_{i=1}^{k}{\varvec{\beta\:}}_{\varvec{i}}{\varvec{X}}_{\varvec{i}}+\sum\:_{\varvec{i}=1}^{\varvec{j}-1}\sum\:_{\varvec{j}=1}^{\varvec{k}}{\varvec{\beta\:}}_{\varvec{i}\varvec{j}}{\varvec{X}}_{\varvec{i}}{\varvec{X}}_{\varvec{j}}+\sum\:_{\varvec{i}=1}^{\varvec{k}}{\varvec{\beta\:}}_{\varvec{i}\varvec{i}}{\varvec{X}}_{\varvec{i}}^{2}$$

Where Y is the system response value; β0, βi, and βii are the offset item, linear offset, and second order offset coefficients, respectively; βij is the interaction coefficient; and Xi is the value of every variable.

**Table 2:** Factors and levels of the Box–Behnken statistical design.

Variables	Unit	Symbol	Level		
			− 1	0	+ 1
Yeast extract	g/L	X_1_	2	5	8
Reaction temperature	°C	X_2_	30	35	40
Culture age	day	X_3_	6	9	12

### Application of the myco-synthesized iron oxide nanoparticles for wastewater remediation

Myco-synthesized IONPs were tested for their capacity to remove five important heavy metals (copper, iron, manganese, zinc, and chromium) from synthetic wastewater. The examined heavy metal ions were prepared at 25 mg/L utilizing their salts. 100 mg/10 mL of IONPs (myco-synthesized by *Aspergillus niger* AUMC 16028) were used for removing these ions from the solutions. Additionally, industrial wastewater samples were examined. These samples were collected from the industrial region of Kafr El-Zayat, El-Gharbia Governorate in Egypt, which had high levels of heavy metals. 100 mg of IONPs were added to 10 mL of the collected wastewater sample and incubated overnight with shaking at 150 rpm^54^. Following that, the heavy metals were measured by an inductively coupled plasma-optical emission spectrometer (ICP-OES, Thermo, iCAP 6000), and the percentage of removal was estimated using Eq. (4):

4 $$\:RE\:\left(\%\right)=\left(\frac{{c}_{i}-{c}_{f}}{{c}_{i}}\right)\times\:100$$

Where RE represents metal removal efficiency; C_i_ is initial concentration of heavy metal (mg/L), and C_f_ is final concentration of heavy metal after treatment (mg/L).

Statistical analysis

All experiments in this study have been replicated three times. The experimental findings were an average value ± SD. The Minitab^®^ 16.1.0 software (Minitab Inc., PA, USA) was used to compare all data using Fisher’s least significant difference (LSD) method and analyze all data using one-way ANOVA.

## Results and discussion

Myco-synthesis of IONPs has several advantages and is extensively employed because they are affordable, non-toxic, and perform a significant role in several geological and biological functions, unlike chemical and physical synthesis. Humans also use them extensively in the production of hemoglobin, catalysts, paints, coatings, long-lasting pigments, colored concrete, and iron ore for thermite^55^. The initial stage in this study was to determine which of the four isolated fungi produced the most myco-IONPs.

### Screening for extracellular production of iron oxide nanoparticles

**Fig. 1:**
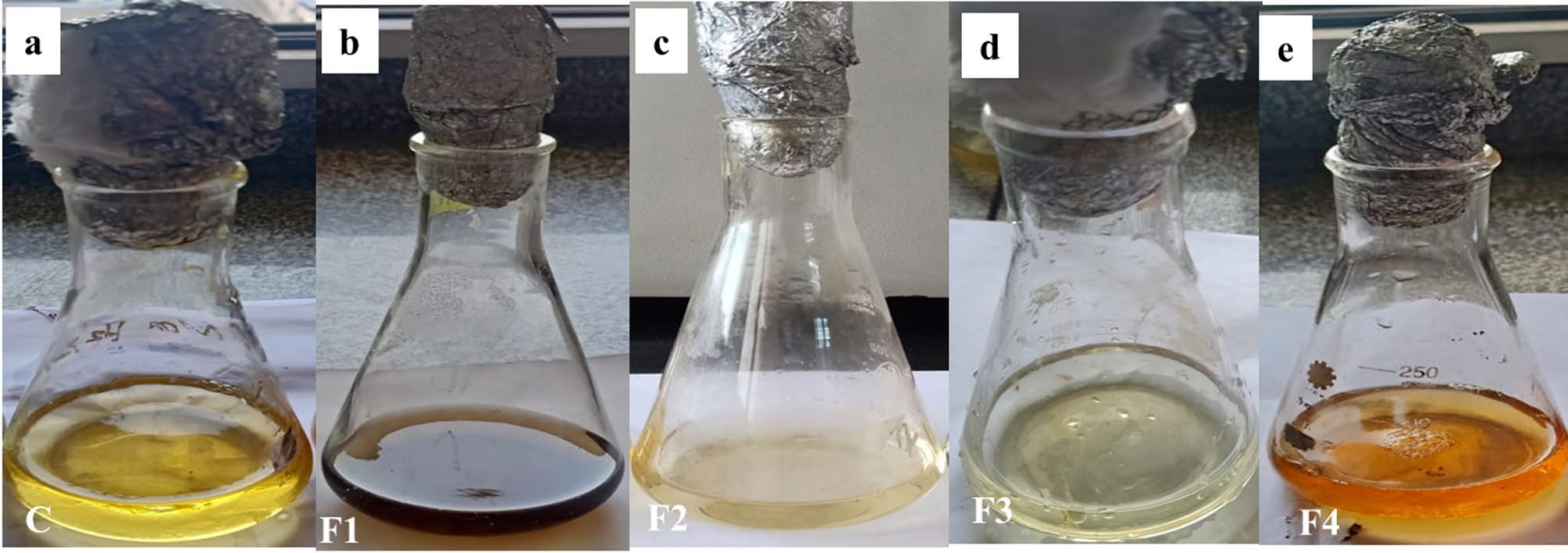
Visual observation of myco-synthesized iron oxide nanoparticles using various mycelial filtrates from tested fungi. **a** is a control; **b**, **c**, **d**, and **e** are the filtrates of the *Aspergillus niger* strain F1, *Aspergillus flavipes* strain F2, *Mucor* sp. strain F3, and *Alternaria* sp. strain F4, respectively.

**Fig. 2:**
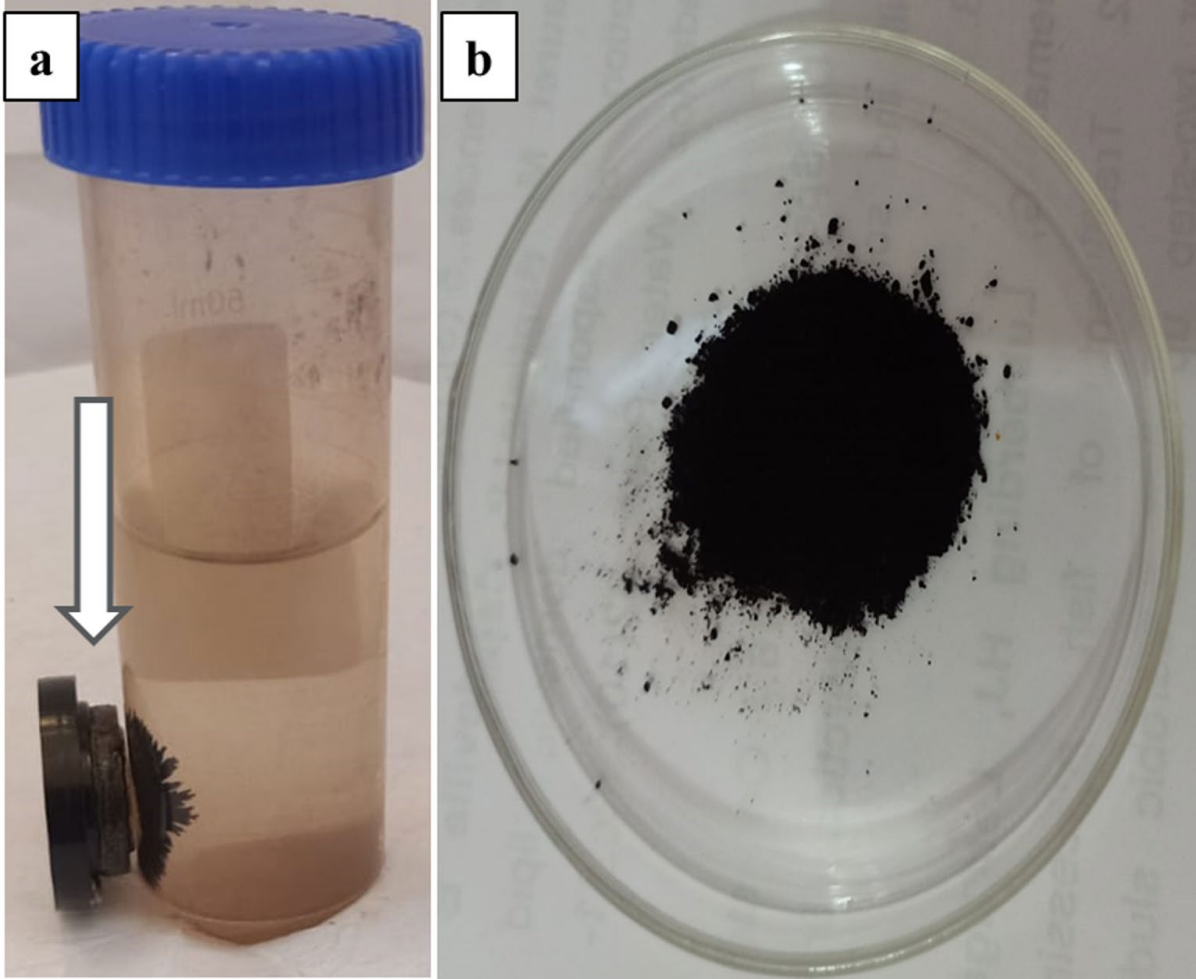
Separation of iron oxide nanoparticles myco-synthesized by *Aspergillus niger* from the mixture reaction solution by external magnet (**a**), the purified powder of iron oxide nanoparticles (**b**).

Color change observation is a typical approach for assessing microbiological isolates to produce IONPs. Extracellular myco-synthesis of IONPs was tested by utilizing each fungus’ mycelial filtrate and detecting color changes in the existence of metal precursors, as seen in Fig. 1. The screening indicated that F1 was the most effective Myco-IONP producer, with samples changing color from yellowish brown to brownish black. This will provide the first insight into IONPs formation. NPs might be removed from the solution by applying an external magnetic field (Fig. 2). Because iron oxide has a distinct surface plasmon resonance feature, which causes the color change^56^. Interaction of metal ions with biologically active chemicals and the surface plasmon resonance that causes this characteristic color shifting^57^. The findings were consistent with those of Saied et al.^58^, who observed that IONPs biosynthesis was accompanied by a color shift to brownish black. This study’s findings are consistent with previous reports of Das et al.^59^, which found that fungi can synthesize enormous quantities of enzymes and proteins utilized in metal bioreduction, leading to the production of biocompatible nanoparticles with minimal complexity in the instrumentation used. Fungal reducing enzymes have been found to perform a vital role in NPs production. Both static and agitated cultures excrete these metabolites outside the cell and into the body solution^60^.

This result was scanned spectrophotometrically from 200 to 800 nm as part of the first validation. The UV-Vis spectroscopy-based technique has been demonstrated to be a valuable tool for monitoring NPs synthesis and characterized as an effective method for the early description of produced metal NPs. Also, this technology has been utilized to observe the composition and stability of metallic NPs, which have great extinction efficiency and bright colors that may be noticed with the naked eye. Therefore, the results obtained contain qualitatively reliable information about the nanoparticles^61^. Based on our findings, isolate F1 was chosen since it was the most effective producer of myco-IONPs and was employed for the remainder of the study.

### Identification of the most effective isolate for producing ionps

**Fig. 3:**
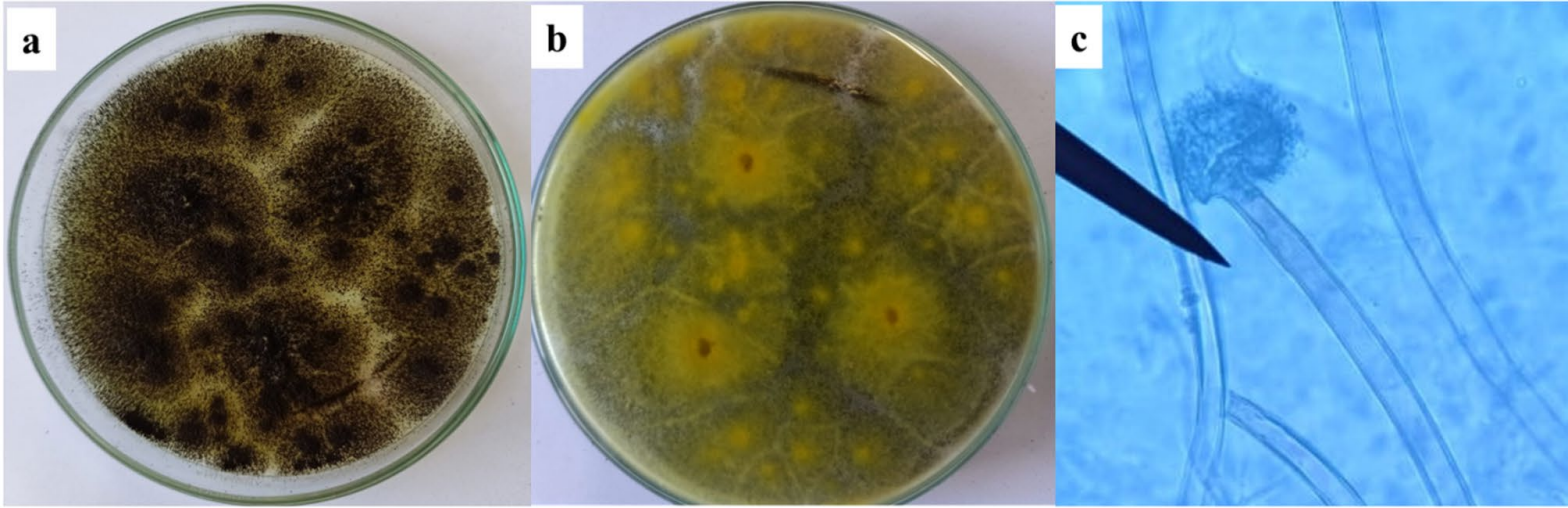
Cultural (**a,b**) and microscopic examination of *Aspergillus niger* under a light microscope at ×40 magnification (**c**).

**Fig. 4:**
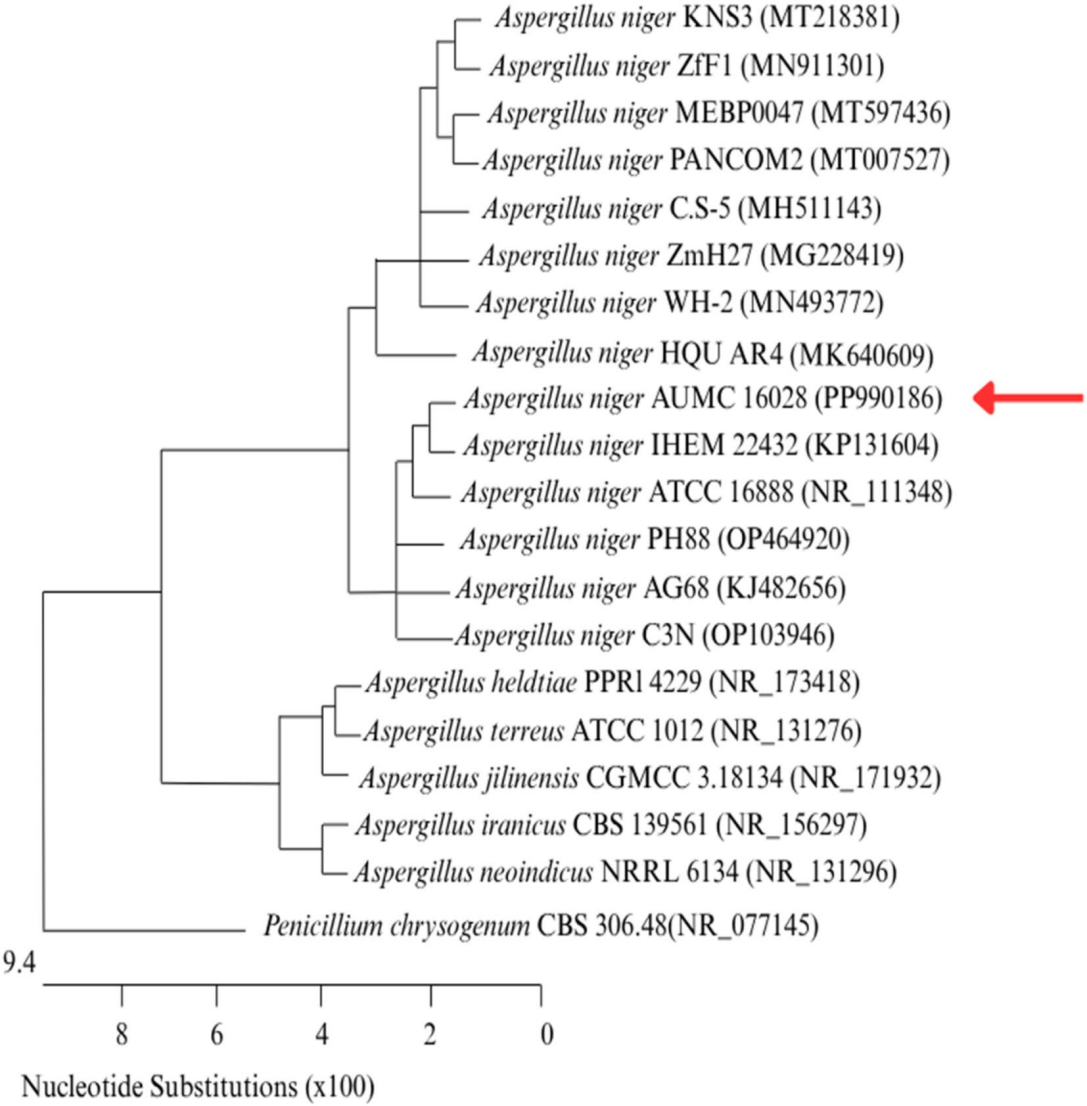
Phylogenetic tree based on ITS sequences of rDNA of *Aspergillus niger* AUMC 16028 (arrowed) aligned with closely related strains obtained from the GenBank.

The most effective fungal isolate F1, was subcultured on PDA and subjected to cultural and microscopic identification^39,40^ and it was found that the fungal isolate belonged to the *Aspergillus* species (Fig. 3). The most potent fungus (F1) that produced IONPs was identified molecularly as *Aspergillus niger* AUMC 16028, and it has been deposited in GenBank with the accession number PP990186. The phylogenetic tree of the selected fungus has been displayed in Fig. 4. This method is regarded as the best way to identify microorganisms^62^.

### Characterization of iron oxide nanoparticles

#### UV–Visible (UV–Vis) spectroscopic analysis

**Fig. 5:**
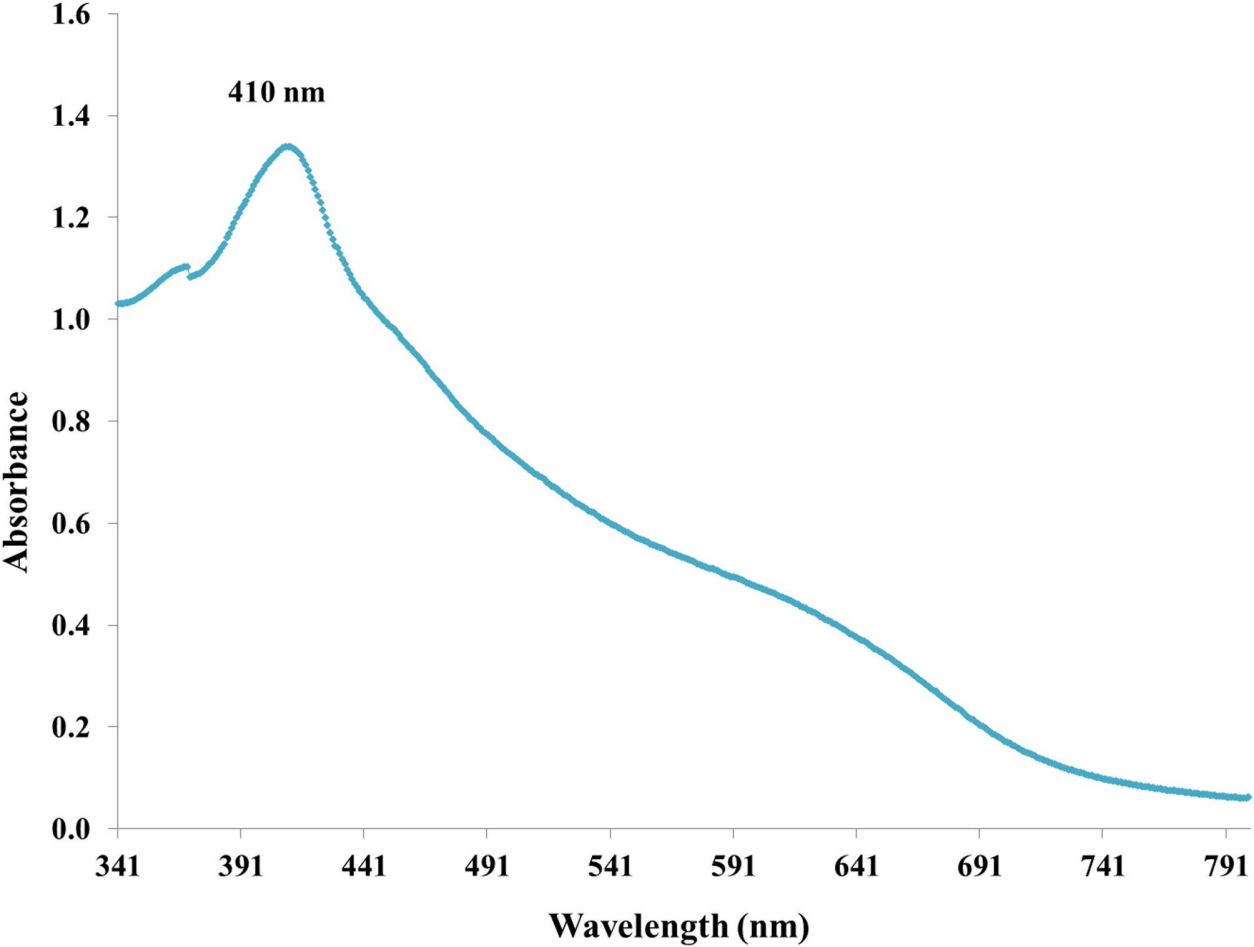
UV-visible spectra showed peaks of iron oxide nanoparticles at 410 nm by cell-free filtrate of *Aspergillus niger* AUMC 16028.

To validate the biosynthesis of IONPs, the sample of *Aspergillus niger* AUMC 16028 was analyzed using UV–vis spectrophotometer examination. IONPs were discovered in the reaction vessel as the reaction mixture shifted from yellowish brown to brownish black throughout the incubation time. The biosynthesized IONPs were assessed by a UV-vis spectrophotometer. The results of this work indicate that the largest peak of the biosynthesized IONPs was seen at 410 nm, as depicted in Fig. 5. In this connection, Siva et al.^63^ used *A. niger* to synthesize cellulase/iron oxide magnetic nanocomposites, where the nanoparticles exhibited a prominent UV–Vis absorption around 400 nm. Similar results were observed by Ali and Alkhafaji^64^. They detected IONPs’ absorption peak between 400 and 500 nm. Also, Saied et al.^58^ detected the myco-synthesis of IONPs using *Aspergillus niger*,* which* had absorption peaks at 490 nm. IONPs were found to produce a surface plasmon resonance spectroscopic signature for the peaks^65^. It is important to note that these findings are consistent with those obtained in previous studies^66^. Also, Carter^67^ found that metal nanoparticle solutions have been observed to exhibit different color features and corresponding peaks.

#### Scanning electron microscopy (SEM)

**Fig. 6:**
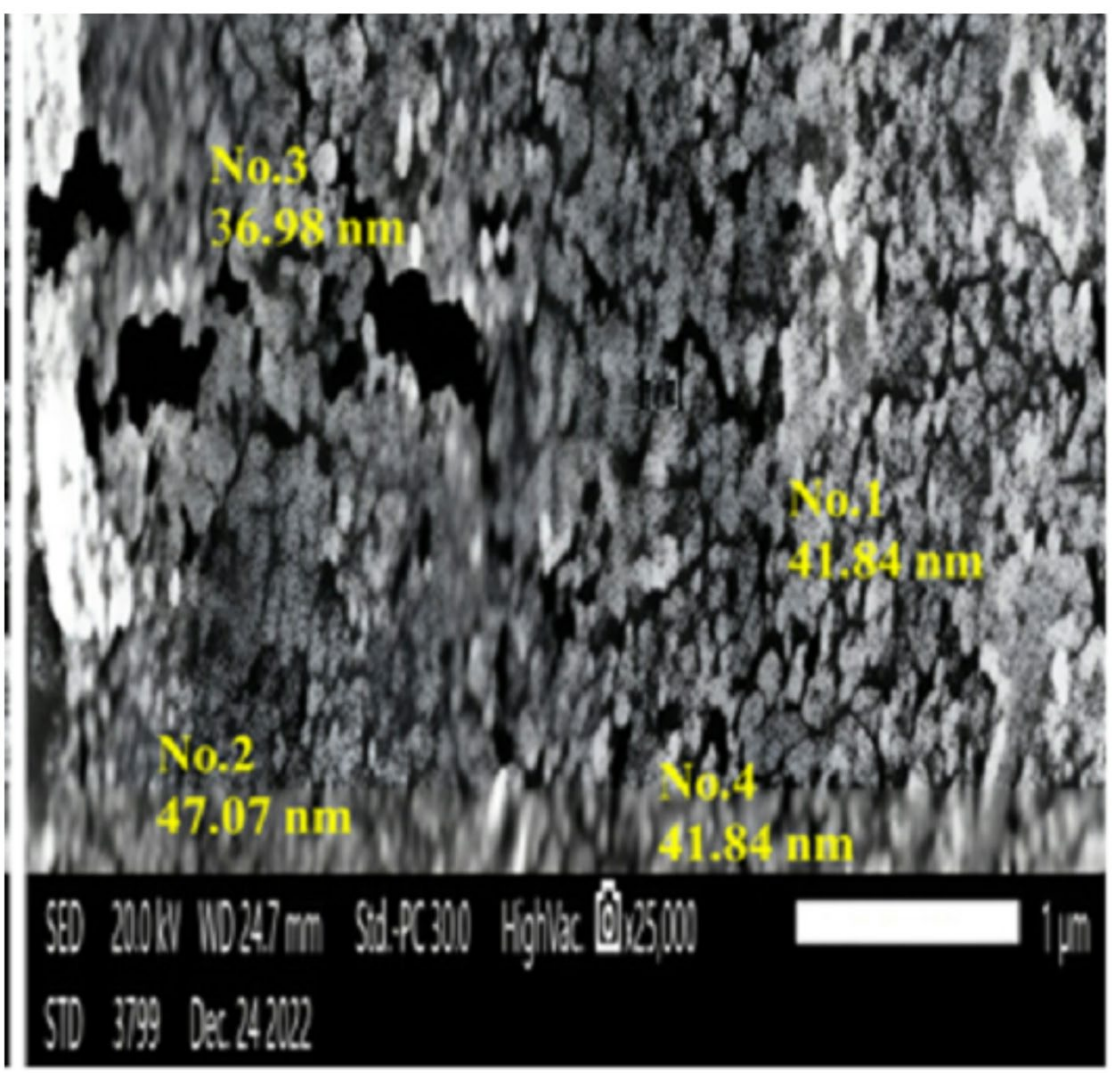
Scanning electron microscopy images of myco-synthesized iron oxide nanoparticles produced in *Aspergillus niger* AUMC 16028 cell-free filtrate.

SEM was used to validate the particle size, surface structure, and image size of IONPs. Biogenic IONPs varied in size from 36 to 47 nm, as shown in Fig. 6. The SEM investigation revealed that NPs had a surface porosity and polydisperse distribution, which enabled molecules to move freely^68^. The nanoparticles aggregated into asymmetrical clusters. Capping agents that covered the surface of the nanoparticles caused the cluster to form^69^.

#### Fourier-transform infrared spectroscopy (FTIR)

**Fig. 7:**
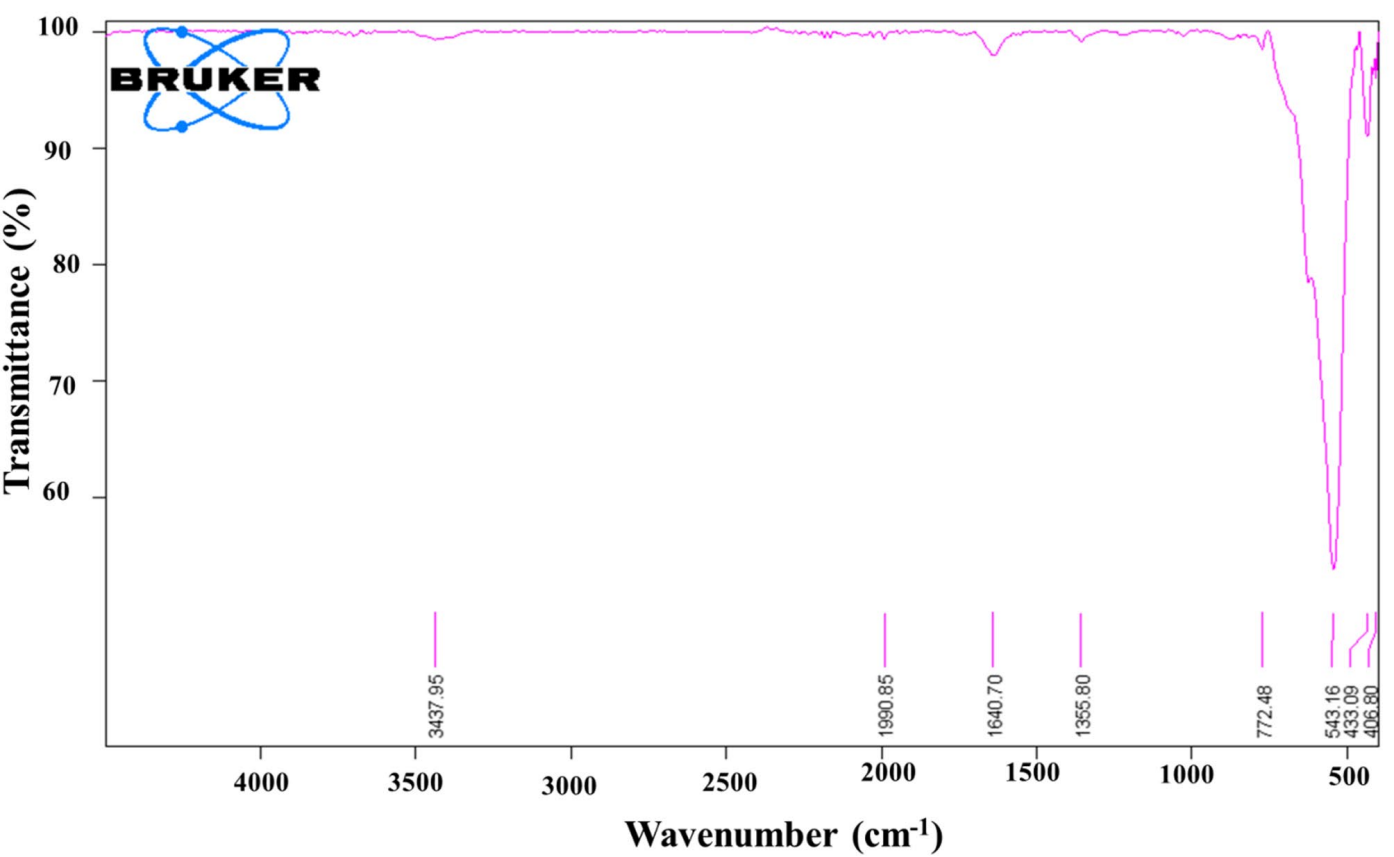
Fourier-transform infrared spectroscopy spectra of iron oxide nanoparticles myco-synthesized by *Aspergillus niger* AUMC 16028.

Fourier-transform infrared spectroscopy (FTIR) spectrum was obtained by a Bruker-8 FT/IR spectrophotometer. This method involves mixing KBr powder with nanoparticles of synthetic iron oxide, which are then quantified in compressed pills. The spectrum was recorded within the 400–4000 wavenumber range. The synthetic nanoparticles FTIR spectra presented in Fig. 7 absorption bands were detected at 3437, 1990, 1640, 1355, 772, 534, 433, and 406 cm⁻¹. The stretching vibrations of the O-H group created the visible peak at 3437 cm⁻¹ due to the postulated presence of flavonoids, phenols, and alcohols^70,71^. Protein amide peaks at 1990 and 1640 cm⁻¹ show broad C = O stretching, probably attributed to the N-H bending vibration of carbonyl β unsaturated ketone amide and secondary amines (protein and lipids)^72^. The peak at 1355 cm⁻¹ may be connected to the C-N stretching vibrations of aromatic and aliphatic amines^73^. The peak at 772 cm^– 1^ reflects the C-H group (alkane and outside the planar band)^74^. Peaks at 534, 433, and 406 cm⁻¹ were due to the natural stretching vibrations of metal-oxygen bonds, specifically iron oxide. This indicated that the generated nanoparticles were 75% iron oxide^75^.

#### Transmission electron microscopy (TEM) analysis

**Fig. 8:**
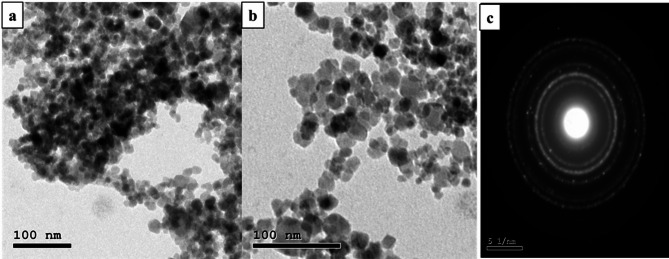
Transmission electron microscopy images of myco-synthesized iron oxide nanoparticles using cell-free filtrate of *Aspergillus niger* AUMC 16028 (**a,b**) and patterns selected area electron diffraction of myco-synthesized iron oxide nanoparticles (**c**).

**Fig. 9:**
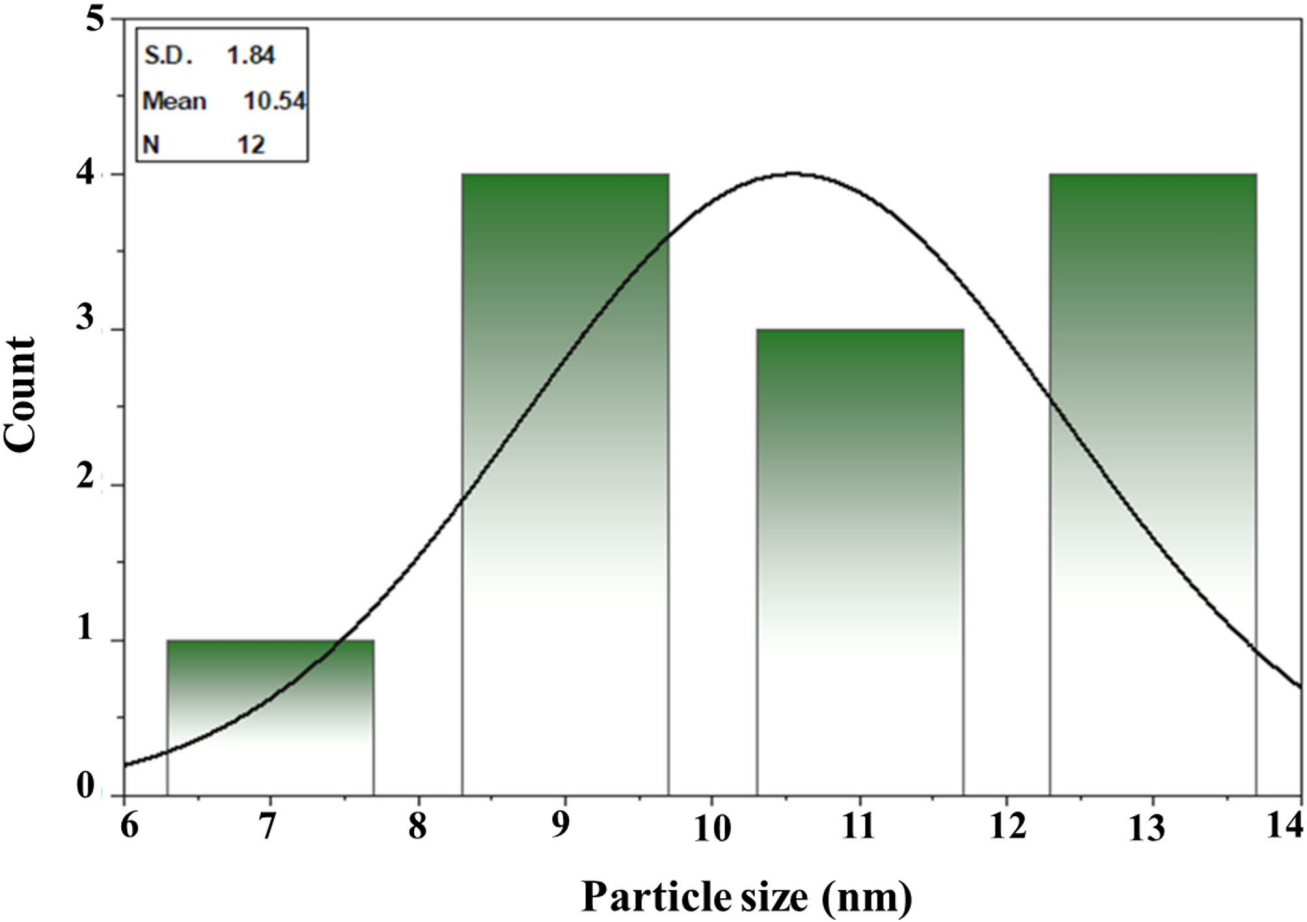
Particle size distribution histogram of myco-synthesized iron oxide nanoparticles.

TEM is an effective method for finding out the final nanoparticles’ form and size^76^. The biosynthesized IONPs in this study were shown to form extracellular spherical and irregular NPs with an average particle diameter of 7 ± 2 nm, as shown in electron micrograph Fig. 8. Sidkey^77^ reported that TEM micrographs of IONPs synthesized from *Aspergillus flavus* D05-F1 showed well-dispersed NPs, spherical particles with an average diameter of 30 ± 3 nm and a size range of 28.6–33.8 nm. Furthermore, Alangari et al.^78^ indicated that IONPs had an average size of roughly 70–100 nm with a spherical form, and some of the NPs were slightly agglomerated, but most of the NPs were evenly distributed. Although most of the particles in the TEM pictures were below 100 nm in size, a few big particles were discovered, which may have been caused by the aggregation of smaller particles. Yassin et al.^79^ found that the biological IONPs had an average particle size of 46.3 nm with a spherical form. Darwesh et al.^80^ discovered that the microscopic images produced demonstrated the formation of nanoparticles from *Fusarium oxysporum* culture growth supernatant. They varied in size from one to three nanometers. The shape of the iron particles was spherical, with minor variations in some aggregates. Our results using ImageJ software showed that the standard deviation of Myco-IONPs was 10.54 nm with an average particle diameter of 1.84, as indicated in Fig. 9.

#### X-ray diffraction spectroscopy analysis

**Fig. 10:**
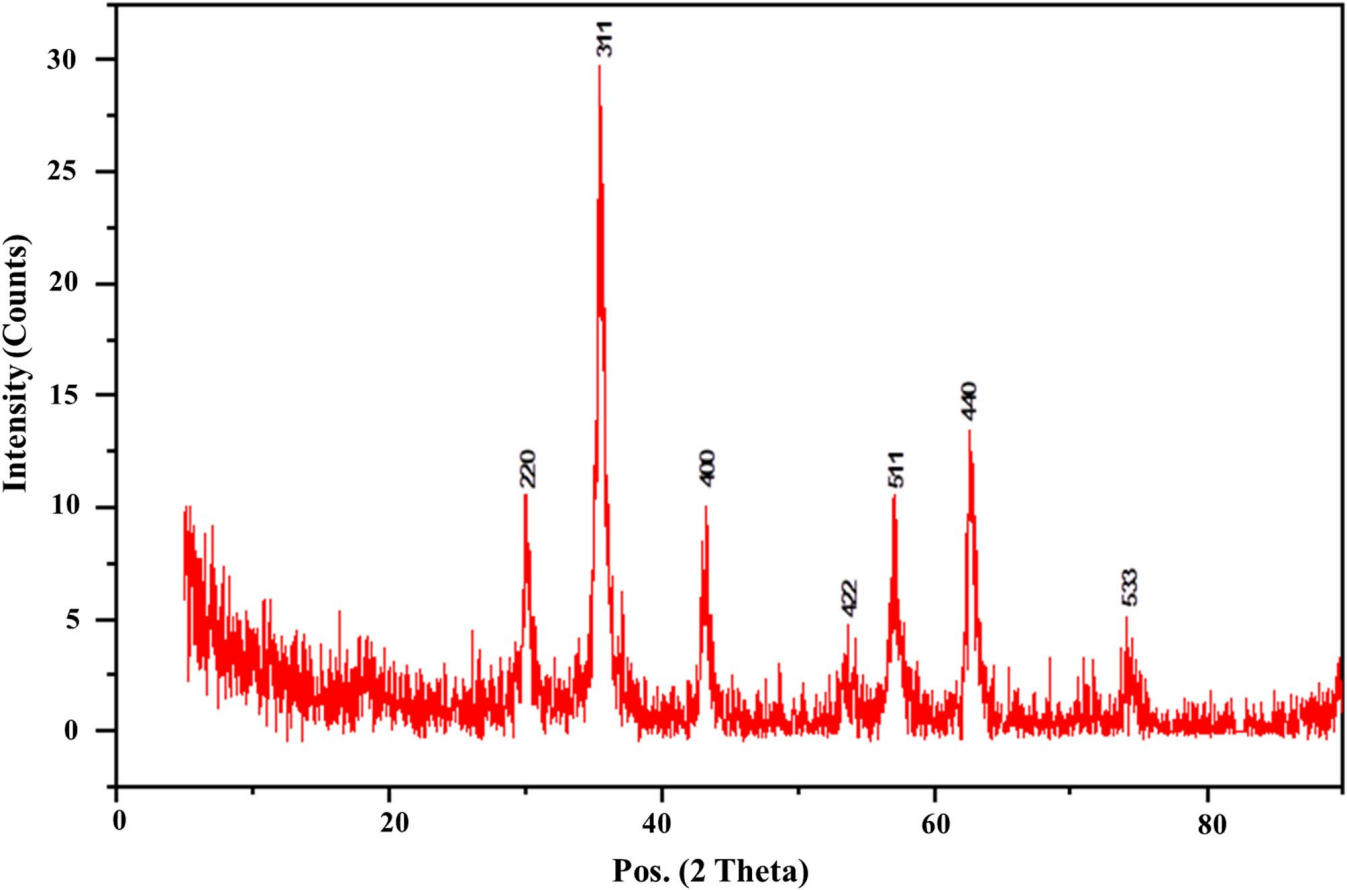
X-ray diffraction spectroscopy of iron oxide nanoparticles myco-synthesized by *Aspergillus niger* AUMC 16028.

Crystal structure and the composition of the prepared samples were determined using X-ray diffraction spectroscopy (XRD) patterns. One crucial method for identifying crystalline formations at the atomic level is XRD^81^. It is a non-destructive method that appears to be promising for characterizing crystalline materials of both biological and inorganic origin. X-ray diffusion is based on Bragg’ law, which indicates that X-rays are elastically scattered at large angles^82^. The XRD pattern is represented in Fig. 10. The XRD data confirms that the primary IONPs powder is in a pure phase (JCPDS Card No. 75–0449). The as-prepared IONPs, matching the cubic inverse spinel structure identified in the JCPDS database, display seven characteristic diffraction peaks equivalent to the crystal planes of (220), (311), (400), (422), (511), (440), and (533) at 2-theta equal to 30.36°, 35.76°, 43.47°, 53.94°, 57.51°, 63.16°, and 74.76°. The Debye-Scherrer equation was used to determine the crystallite size, which was 8.73 nm. This was consistent with the outcome shown in Fig. 8, which was the TEM picture. Magnetite particles have an average diameter of 7 ± 2 nm and are spherical, as seen in this figure. These outcomes agree with the iron oxide nanoparticles that were previously created via biological processes^66^.

#### Energy dispersive x-ray analysis

**Fig. 11:**
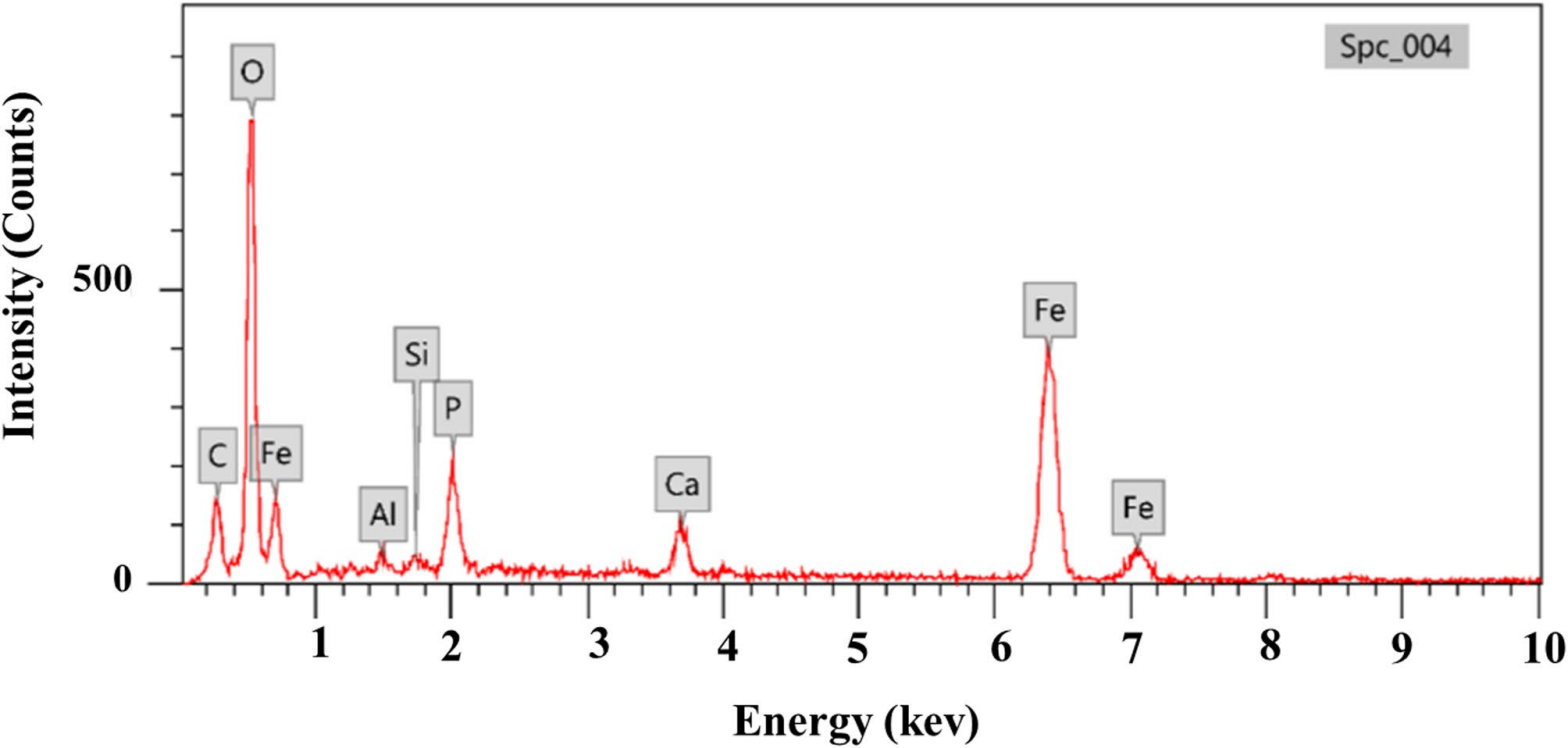
Energy dispersive x-ray characterization of iron oxide nanoparticles derived from *Aspergillus niger* AUMC 16028.

The myco-synthesized IONPs’ elemental composition was characterized using energy dispersive x-ray (EDAX); the resulting image and analysis are shown in Fig. 11. By observing the carbon spectrum, the theory that organic moiety plays a crucial function as a capping agent was confirmed. The weight percentages of Fe, O, C, Ca, P, Si, and Al were 34.78, 41.21, 14.84, 2.89, 2.98, 5.02, 0.42, and 0.75, respectively. The hypothesis that the organic fraction plays an important role as a covering agent was confirmed by measuring the carbon spectrum. The presence of calcium, phosphorus, silicon, and aluminum indicates impurities. Elemental iron was present, as seen by the strong peaks at 6 and 7 keV. The oxidation of nanoparticles because of contact with air and water was revealed by the oxygen spectrum^83^.

### Optimization of myco-synthesized iron oxide nanoparticles

#### Plackett-Burman experimental results and analysis

**Fig. 12:**
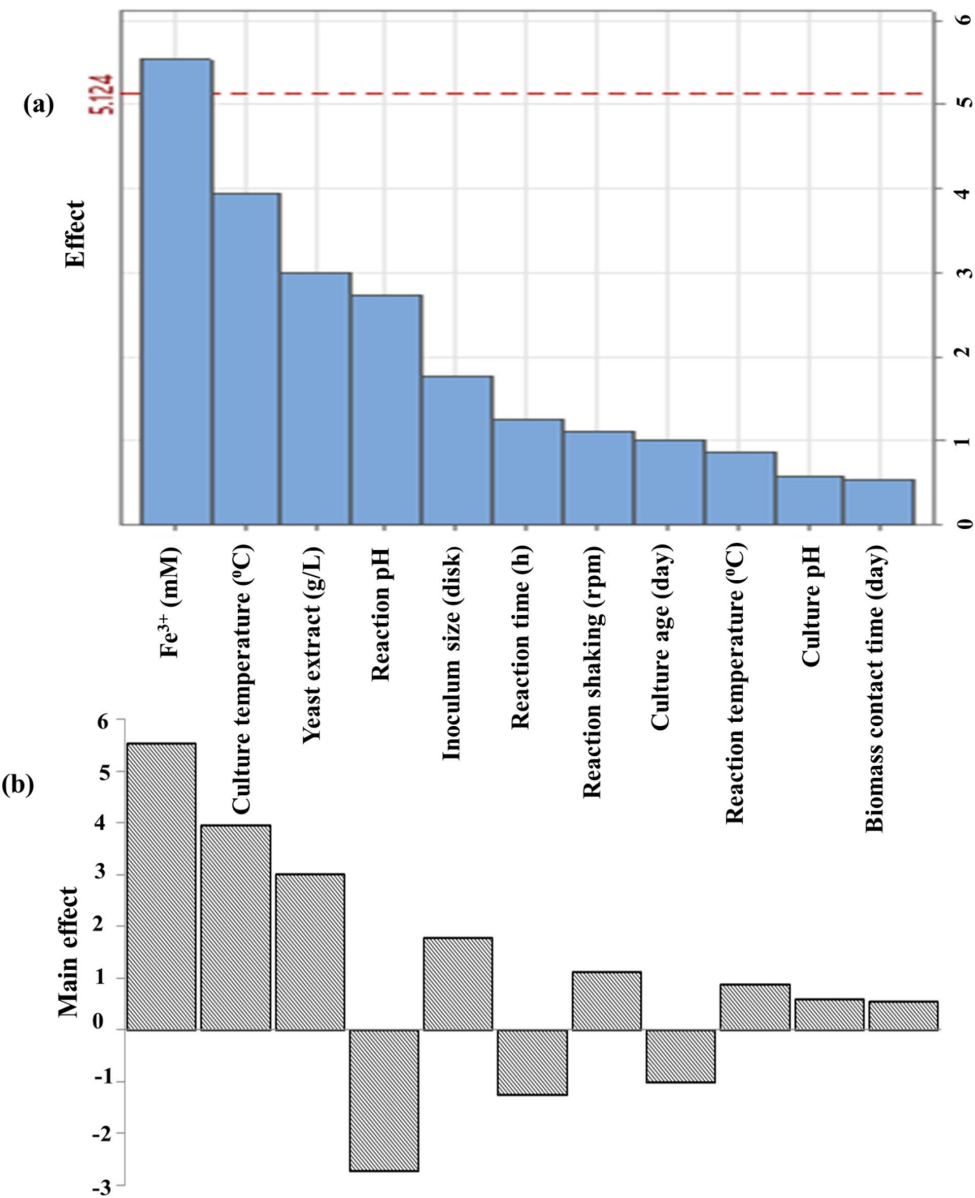
Pareto chart (**a**) and main effects (**b**) of ferric removal percentage by iron oxide nanoparticle myco-synthesized using the Plackett-Burman design.

**Fig. 13:**
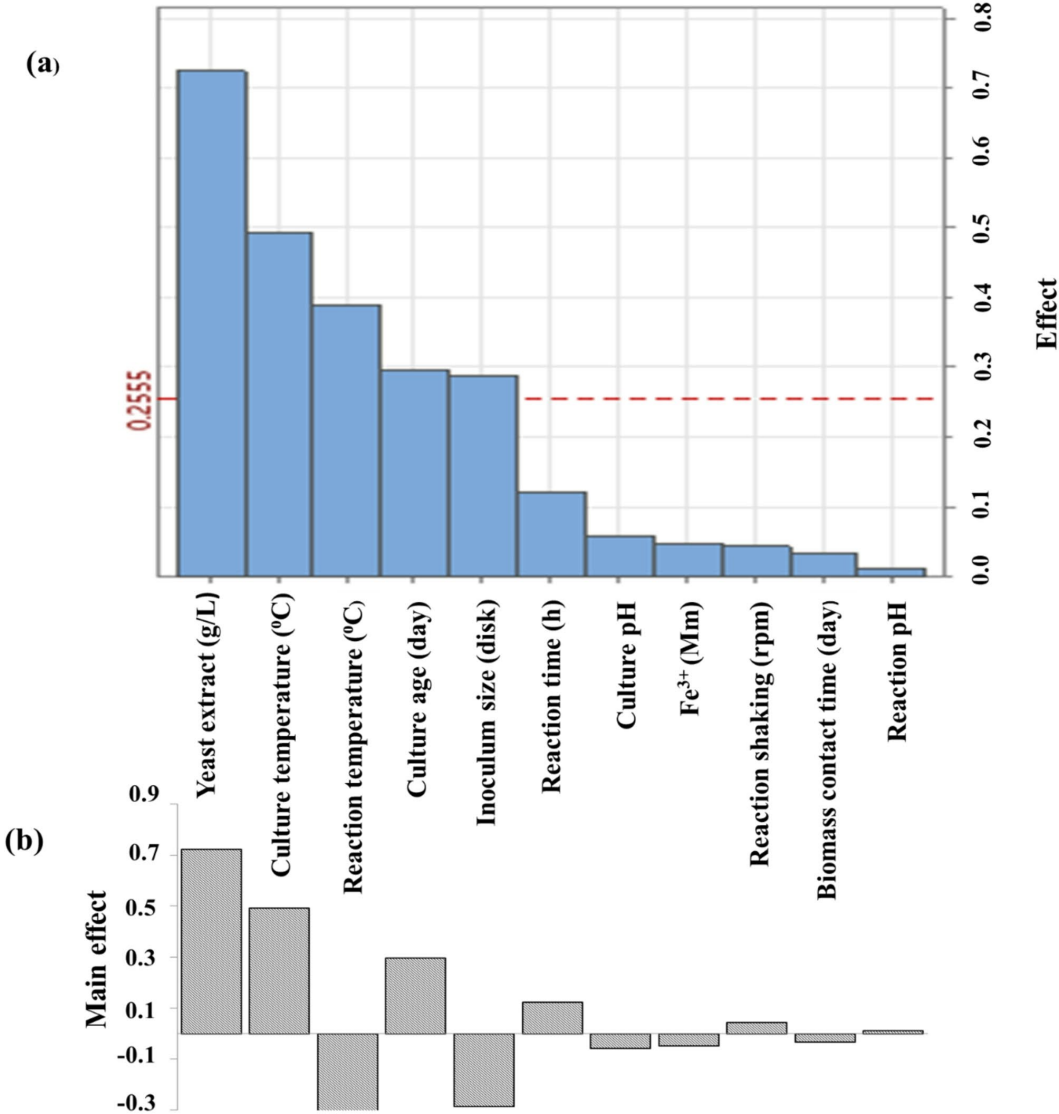
Pareto chart (**a**) and main effects of OD_410nm_ (**b**) via iron oxide nanoparticles myco-synthesized through Plackett-Burman design.

Plackett-Burman design (PBD) is significantly faster and easier to use than other statistical methods^84^. Traditional optimization techniques disregard interactions between independent factors and doing several trials is expensive and time-consuming. Statistical experimental design was used to assess the effect of various parameters on the production of biomass and IONPs output. Eleven variables were chosen in PBD to represent the most important physiological and environmental factors influencing myco-synthesis of IONPs using fungal isolate (Table 3). Twelve assays were made as stated by PB design, and the removal percentage of iron and optical density were obtained as given in Table 3. According to the table, trials 3, 8, and 11 had the highest iron removal rates (99.16, 99.45, and 99.71%), while trial 1 had the lowest iron removal rate (86.49%). Trial 5 also had the highest optical density (1.4 nm), while trial 12 had the lowest (0.08 nm). Figures 12 and 13 show the mean values of the main effect of the tested factors on myco-synthesis of IONPs. The Pareto chart is ideal for displaying the findings of a PB design, as it indicates the magnitude of each factor estimate regardless of its impact, whether positive or negative^85^. The highest positive significant variable was Fe^3+^, affecting the removal of iron, while the highest positive significant variables affecting the optical density were yeast extract, culture temperature, reaction temperature, and culture age.

A positive effect indicates that a better reaction was obtained when a higher level of concentration was utilized, whereas a negative effect indicates that better results are favored at lower concentrations. Figures 12 and 13 present a handy approach to observe the findings of PBD by providing the Pareto chart of the effects, which displays the relative significance levels.

**Table 3:** Plackett-Burman design matrix for independent factors affecting iron oxide nanoparticles myco-synthesis.

Std order	Run order	Pt type	Blocks	Culture age (day)	Culture temperature (⁰C)	Culture pH	Yeast extract (g/L)	Inoculum size (disk)	Biomass contact time (day)	Reaction time (h)	Reaction temperature (⁰C)	Reaction pH	Fe^3+^ (mM)	Reaction shaking (rpm)	Removal of Fe^3+^ (%) ± SD	OD_410nm_ ± SD
1	1	1	1	1(9)	− 1(25)	1(8)	− 1(0)	− 1(1)	− 1(1)	1(48)	1(35)	1(8)	− 1(1)	1(150)	86.49 ± 1.43	0.10 ± 0.002
2	2	1	1	1(9)	1(35)	− 1(5)	1(5)	− 1(1)	− 1(1)	− 1(24)	1(35)	1(8)	1(3)	− 1(0)	98.55 ± 0.89	1.17 ± 0.004
3	3	1	1	− 1(5)	1(35)	1(8)	− 1(0)	1(2)	− 1(1)	− 1(24)	− 1(25)	1(8)	1(3)	1(150)	99.16 ± 0.57	0.23 ± 0.002
4	4	1	1	1(9)	− 1(25)	1(8)	1(5)	− 1(1)	1(3)	− 1(24)	− 1(25)	− 1(5)	1(3)	1(150)	98.70 ± 0.82	1.00 ± 0.002
5	5	1	1	1(9)	1(35)	− 1(5)	1(5)	1(2)	− 1(1)	1(48)	− 1(25)	− 1(5)	− 1(1)	1(150)	96.50 ± 2.29	1.47 ± 0.002
6	6	1	1	1(9)	1(35)	1(8)	− 1(0)	1(2)	1(3)	− 1(24)	1(35)	− 1(5)	− 1(1)	− 1(0)	95.65 ± 1.63	0.10 ± 0.001
7	7	1	1	− 1(5)	1(35)	1(8)	1(5)	− 1(1)	1(3)	1(48)	− 1(25)	1(8)	− 1(1)	− 1(0)	93.03 ± 2.3	1.34 ± 0.001
8	8	1	1	− 1(5)	− 1(25)	1(8)	1(5)	1(2)	− 1(1)	1(48)	1(35)	− 1(5)	1(3)	− 1(0)	99.45 ± 0.42	0.14 ± 0.008
9	9	1	1	− 1(5)	− 1(25)	− 1(5)	1(5)	1(2)	1(3)	− 1(24)	1(35)	1(8)	− 1(1)	1(150)	93.50 ± 2.04	0.15 ± 0.001
10	10	1	1	1(9)	− 1(25)	− 1(5)	− 1(0)	1(2)	1(3)	1(48)	− 1(25)	1(8)	1(3)	− 1(0)	91.78 ± 0.82	0.14 ± 0.008
11	11	1	1	− 1(5)	1(35)	− 1(5)	− 1(0)	− 1(1)	1(3)	1(48)	1(35)	− 1(5)	1(3)	1(150)	99.71 ± 0.24	0.27 ± 0.001
12	12	1	1	− 1(5)	− 1(25)	− 1(5)	− 1(0)	− 1(1)	− 1(1)	− 1(24)	− 1(25)	− 1(5)	− 1(1)	− 1(0)	88.93 ± 2.61	0.08 ± 0.001

Values for the independent variables are between brackets. Response data are displayed as mean ± standard error.

#### Optimization of iron oxide nanoparticles myco-synthesis utilizing Box-Behnken design

To find the optimal response area for myco-synthesis of IONPs, the independent parameters, including yeast extract (X1), reaction temperature (X2), and culture age (X3), were studied using the BBD at three distinct levels (-, 0, and +)^52^. The remaining components of the production mixture were introduced at the optimal concentration determined by PBD and considered as constant variables. Table 4 shows the coded factors’ design matrix in addition to the experimental optical density values. The findings showed that the optical density changed greatly based on the values of the three independent parameters. According to the findings of the statistical study, the empirical link between the response and the yeast extract (X1), reaction temperature (X2), and culture age (X3) may be explained by Eq. (5):

5 $$\begin{aligned} Y&=1.43689-0.02950{X}_{1}+0.06783{X}_{2}-0.03117{X}_{3}+0.05447{X}_{1}{X}_{1}+0.16947{X}_{2}{X}_{2}\\ & \quad +0.01214{X}_{3}{X}_{3}+0.07500{X}_{1}{X}_{2}+0.03600{X}_{1}{X}_{3}-0.06883{X}_{2}{X}_{3}\end{aligned}$$

The ANOVA findings in Table 5 show that the BBD model is significant (F-value = 93.07). However, the lack of fit error is statistically insignificant (p-value = 0.076). The coefficient of determination (R²) is utilized to determine the model’s goodness of fit^86^. In the present study, the correction coefficient (R²) is 0.9941, showing that the model is highly correlated. The corrected correction coefficient (adjusted R²) is 0.9834, which means that the regression model may replicate 98.34% of the changes in the experimental data. When calculating the modified R² value, the number of terms and sample size in the model might be considered as variables.

With one variable held constant at its optimal level and the other two variables varied within the experimental range, the regression equation is represented graphically by the three-dimensional response surface plots, which are used to calculate the myco-synthesis of IONPs for each pairwise combination of the three factors. The hump in the three-dimensional graphic was used to determine each factor’s ideal value.

**Fig. 14:**
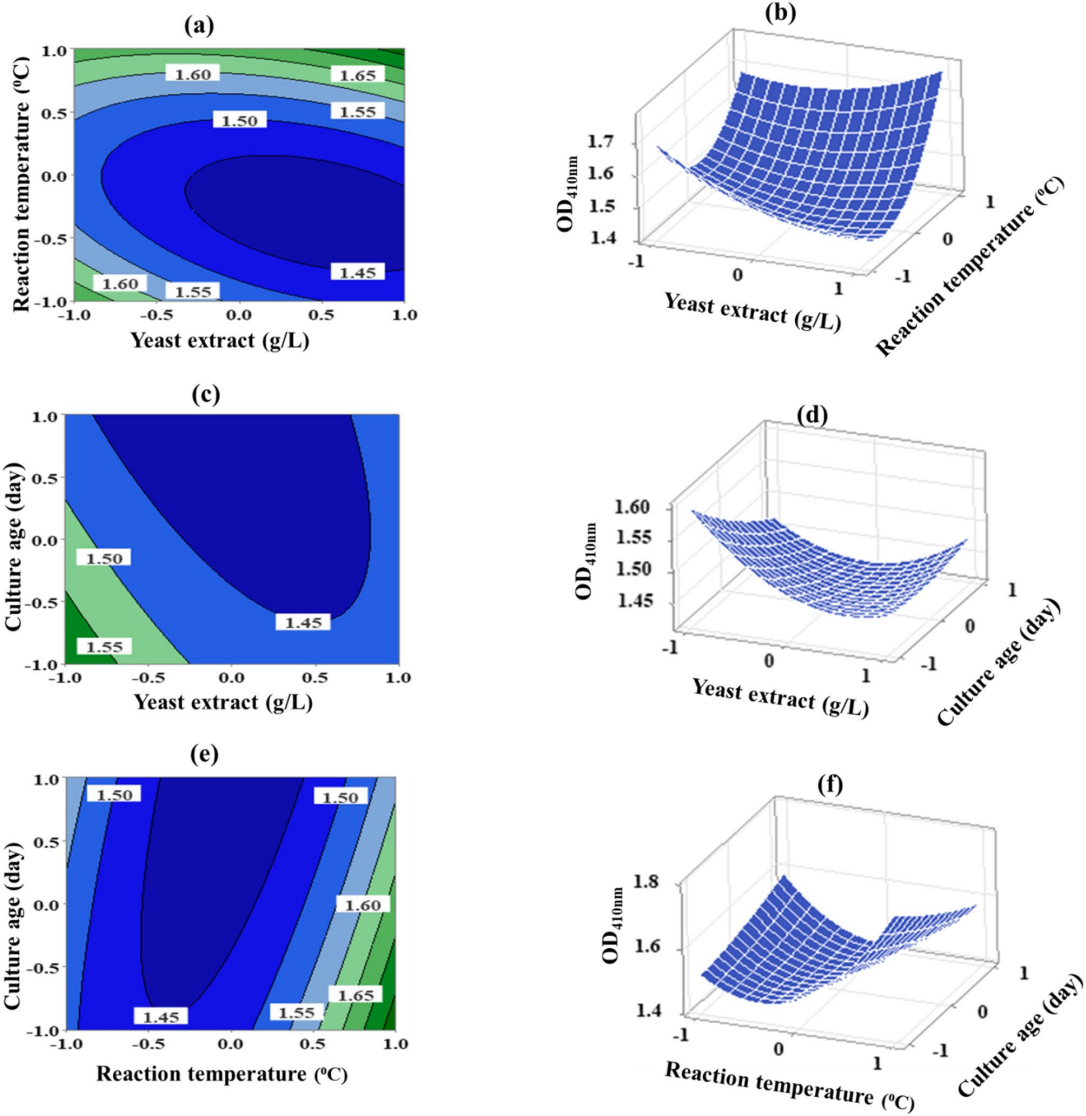
Three-dimension response surface and two-dimension contour plots indicating the effects of reaction temperature and yeast extract (**a,b**), culture age and yeast extract (**c,d**), culture age and reaction temperature (**e,f**) on the formation of iron oxide nanoparticles at optical density 410 nm.

It was easier to understand the interacting effects of the factors under study when the findings were shown as a surface plot. Figure 14 depicts a surface plot of independent variables influencing IONPs myco-synthesis according to the findings of the BBD. (a, b) reflects the interaction between reaction temperature and yeast extract, (c, e) reflects the interaction between culture age and yeast extract, and (d, e) reflects the interaction between culture age and reaction temperature. The results below indicate that the optimal conditions for myco-synthesis of IONPs were reached at independent variable values that were extremely close to the basal levels.

**Fig. 15:**
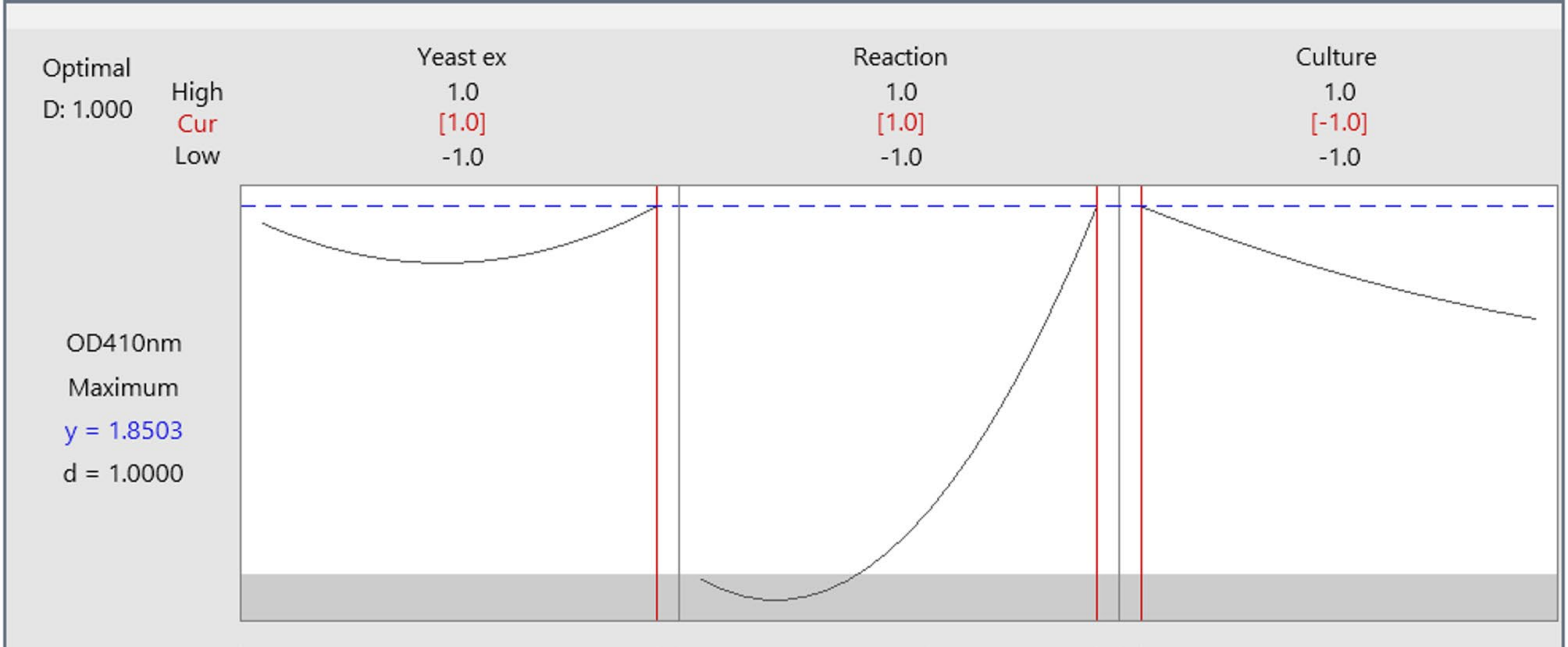
Optimum process parameters for the highest myco-synthesized oxide nanoparticles.

The response optimizer program was used to create optimization curves. Figure 15 shows the final optimal values for the parameters that maximize IONPs synthesis. Optimal conditions for IONPs myco-synthesis include yeast extract (8 gm), reaction temperature (40 °C), and culture age (6 days).

**Table 4:** Factors affecting iron oxide nanoparticles formation according to Box-Behnken design.

Std order	Run order	Pt type	Blocks	Yeast extract (g/L)	Reaction temperature (°C)	Culture age (days)	OD_410 nm_ ±SD
1	1	2	1	− 1(2)	− 1(30)	0(9)	1.686 ± 0.001
2	2	2	1	1(8)	− 1(30)	0(9)	1.486 ± 0.003
3	3	2	1	− 1(2)	1(40)	0(9)	1.686 ± 0.002
4	4	2	1	1(8)	1(40)	0(9)	1.786 ± 0.002
5	5	2	1	− 1(2)	0(35)	− 1(6)	1.596 ± 0.002
6	6	2	1	1(8)	0(35)	− 1(6)	1.456 ± 0.003
7	7	2	1	− 1(2)	0(35)	1(12)	1.479 ± 0.004
8	8	2	1	1(8)	0(35)	1(12)	1.483 ± 0.001
9	9	2	1	0(5)	− 1(30)	− 1(6)	1.529 ± 0.005
10	10	2	1	0(5)	1(40)	− 1(6)	1.788 ± 0.005
11	11	2	1	0(5)	− 1(30)	1(12)	1.587 ± 0.002
12	12	2	1	0(5)	1(40)	1(12)	1.570 ± 0.000
13	13	0	1	0(5)	0(35)	0(9)	1.443 ± 0.003
14	14	0	1	0(5)	0(35)	0(9)	1.435 ± 0.005
15	15	0	1	0(5)	0(35)	0(9)	1.433 ± 0.003

**Table 5:** Analysis of variance (ANOVA) of three factors (yeast extract, reaction temperature, and culture age) of iron oxide nanoparticles produced by *Aspergillus Niger* AUMC 16028.

Source	DF	Adj SS	Adj MS	F-value	*P*-value
Model	9	0.210659	0.023407	93.07	0.0001
Linear	3	0.051544	0.017181	68.32	0.0001
Yeast extract	1	0.006962	0.006962	27.68	0.003
Reaction temperature	1	0.036811	0.036811	146.38	0.0001
Culture age	1	0.007771	0.007771	30.90	0.003
Square	3	0.112479	0.037493	149.09	0.0001
Yeast extract*Yeast extract	1	0.010956	0.010956	43.57	0.001
Reaction temperature * Reaction temperature	1	0.106046	0.106046	421.69	0.0001
Culture age * Culture age	1	0.000544	0.000544	2.16	0.201
2-Way Interaction	3	0.046636	0.015545	61.82	0.0001
Yeast extract*Reaction temperature	1	0.022500	0.022500	89.47	0.0001
Yeast extract*Culture age	1	0.005184	0.005184	20.61	0.006
Reaction temperature*Culture age	1	0.018952	0.018952	75.36	0.0001
Error	5	0.001257	0.000251		
Lack-of-fit	3	0.001193	0.000398	12.37	0.076
Pure error	2	0.000064	0.000032		
Total	14	0.211916			

### Application of the myco-synthesized iron oxide nanoparticles for wastewater remediation

**Fig. 16:**
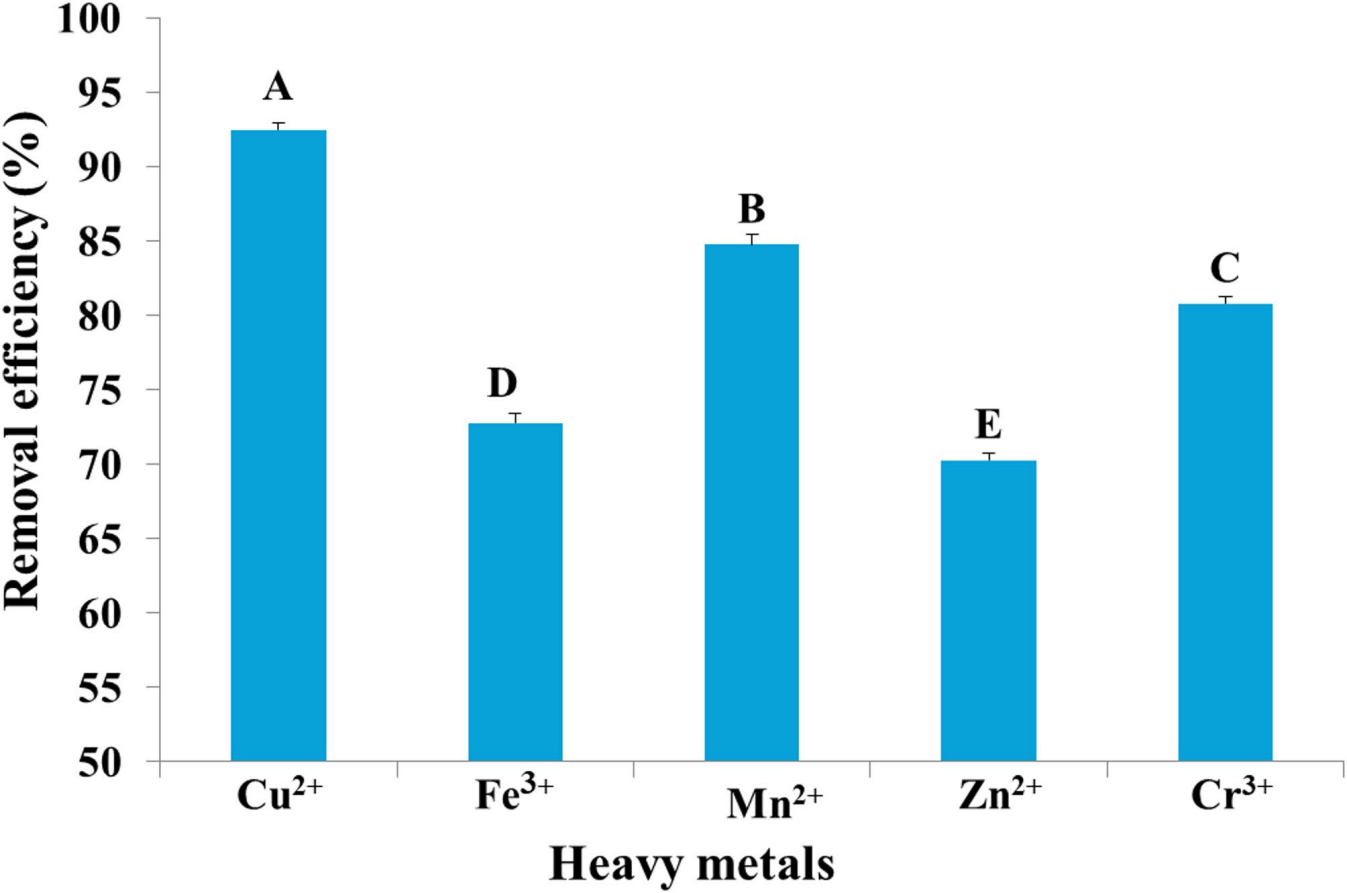
Removal efficiency of copper, iron, manganese, zinc, and chromium from synthetic wastewater by iron oxide nanoparticles myco-synthesized by *Aspergillus niger* AUMC 16028.

**Fig. 17:**
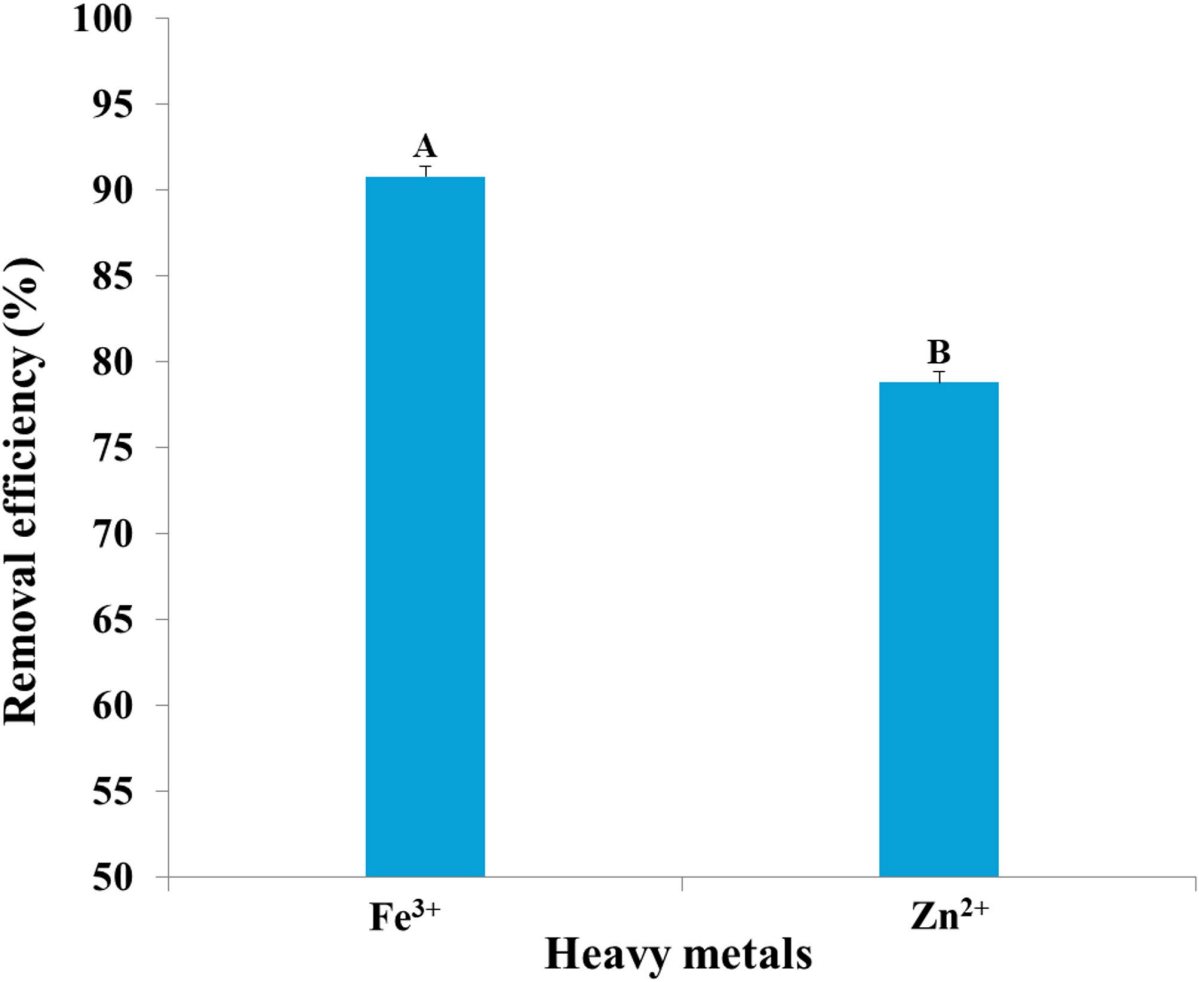
Removal efficiency of the heavy metals iron and zinc from industrial wastewater by iron oxide nanoparticles myco-synthesized by *Aspergillus niger* AUMC 16028.

Myco-synthesized IONPs were evaluated for their capacity to remove heavy metals from synthetic wastewater. After applying 100 mg/10 mL of biosynthesized IONPs to synthetic wastewater for 24 h, the percentages of heavy metal removal were calculated and were shown in Fig. 16. The results indicated that the removal efficiency for heavy metals reached 92.13, 92.49, 84.76, 80.79, and 72.63% for Cu^2+^, Zn^2+^, Mn^2+^, Cr^3+,^ and Fe^3+^, respectively. On the other hand, myco-synthesized IONPs were used to treat industrial wastewater collected from the industrial region of Kafr El-Zayat, El-Gharbia Governorate. Figure 17 shows that IONPs could adsorb heavy metals to nearly 90.30% for Fe^3+^ and 78.44% for Zn^2+^. In accordance with our results, Swelam et al.^87^ revealed that magnetic IONPs may be used for the removal of Fe^3+^ ions from polluted water several times. Magnetic IONPs may be employed more than once to remove Fe^3+^ ions from contaminated water, with a maximum adsorption capacity of 28.225 mg/g and a removal percentage of roughly 85% at an adsorbent dose of 0.22 g. Darwesh et al.^80^ also found that iron nanoparticles have the potential to treat municipal wastewater approximately 95% for lead and proportions ranging from 20 to 50% with the heavy metals cadmium, chromium, nickel, and zinc. In addition, Mohamed et al.^88^ found that the maximal removal ratio of Cr^6+^ ions by iron oxide commercial activated carbon nanocomposite was found to be 98.6% at an adsorbent concentration of 2 g/L, an initial pollutant concentration of 100 mg/L, 3 h of contact time, and room temperature. Also, Khan et al.^89^ found that the removal efficiency of copper ions by IONPs was 94% and Vasile et al.^90^ reported that the use of Fe_3_O_4_ nanomaterial adsorbent for removing Mn (II) ions results in wastewater treatment efficiency higher than 97% at pH 11.5. However, Heavy metals are eliminated by nanoparticles, mostly through adsorption on their surface. The adsorption affinity of IONPs for various heavy metals prevalent in the environment differs depending on the element; hence, the removal effectiveness of different elements varies under similar conditions^91^. Recently, Juturu et al.^92^ demonstrated the efficient adsorption of Cr⁶⁺ using a novel magnetic biochar composite, emphasizing the vital role of magnetic nanomaterials in reducing chromium from wastewater. Moreover, a magnetic perovskite-spinel oxide nanocomposite synthesized for environmental applications through a sol–gel self-combustion process was used as an adsorbent to remove toxic heavy metals^93^.

As a possible new resource for water treatment, our work and other researchers have supported the importance of surface area and the type of pores (meso/micro/macro) in the adsorption of heavy metals and other pollutants by iron-based NPs. In this context, nanomaterials have emerged as attractive materials as wastewater treatment because of their distinct morphological, mechanical, and physicochemical features, which provide them numerous benefits over traditional materials in this regard. Their exceptional qualities such as their ultra-large surface area, superior mechanical strength, and adjustable surface chemistry as well as their polar and non-polar chemistries, controlled and size-tunable properties, and simpler biodegradation have drawn a lot of attention from researchers in recent years^94^. These qualities make them perfect candidates for use in water and environmental remediation applications^94,95^. The remarkable physicochemical qualities of magnetite nanoparticles particularly their strong magnetization, distinctive electrical features, high surface area, compact size, and high adsorption capacity have drawn a lot of interest. The presence of magnetic material in the adsorbents and the ease with which its surface can be functionalized with various surfactants allow them to be readily separated from water by the application of a magnet. Fe_3_O_4_ (MNPs) have been created, coated with various substances, and used for water purification and environmental cleanup. When used to remove Cu^2+^, silica-coated magnetite-NH2 with a mesostructure demonstrated an adsorption capability of 0.5 mmol/g^96,97^. Iron oxides have been extensively researched as effective adsorbents for the elimination of arsenic since they are inexpensive and non-toxic materials. The impact of a meso- (SBA15) and macrostructured (MOSF) silica support’s textural characteristics on the structural-morphological characteristics and adsorption capacity of the active phase (Fe_2_O_3_) has been investigated. Given that relatively small Fe_2_O_3_ NPs (approximately 5 nm) were obtained, the results indicate that mesostructured materials are better suited for dispersing active phases as adsorbents for water treatment. Faster kinetics were demonstrated by the amino-functionalized SBA15 adsorbent (3-aminopropyltriethoxysilane, APTES). Additionally, concerns about the release of silicon and iron during the sorption process, which results in secondary pollution, were assessed and examined severely by^98^.

## Conclusion

Bio nanotechnologies have gained a lot of interest because of their promising findings and potential advantages in several fields of life. To summarize, in this investigation we have successfully synthesized iron nanoparticles (IONPs) using cell-free extract of *Aspergillus niger* strain F1, *A. flavipes* strain F2, *Mucor* sp. strain F3, and *Alternaria* sp. strain F4. and evaluated the efficiency of the most potent fungus in removing heavy metals. The most potent fungus that produced IONPs was identified molecularly as *Aspergillus niger* AUMC 16028. IONPs formation was identified by a color shift using UV-Vis spectroscopy. Scanning electron microscopy (SEM), Fourier-transform infrared spectroscopy spectra (FT-IR), X-ray diffraction spectroscopy (XRD), energy dispersive X-ray (EDAX), and transmission electron microscopy (TEM) were all utilized to characterize the myco-synthesized IONPs. The experiments were performed under controlled conditions. However, Plackett-Burman design (PBD) and Box-Behnken design (BBD) were used to optimize the myco-synthesis of IONPs. The results indicated that myco-synthesized IONPs are efficient in removing the heavy metals copper, iron, manganese, zinc, and chromium from synthetic wastewater and the heavy metals iron and zinc from industrial wastewater effluent. These findings support an effective, economical, and sustainable industrial wastewater bioremediation method. Myco-nanotechnology has lately gained attention due to the potential uses of biosynthesized nanoparticles made by fungi. Future research and development of novel fungal-derived nanoparticles may mark an important milestone in sustainable bioremediation. Their hybrids with other materials need further research. In vivo toxicological models must also be used to assess the pertinent harmful effects of IONPs. To enable useful fungal-mediated IONPs applications in a range of fields, future studies should concentrate on shedding light on existing knowledge gaps within this domain and closing the gap between laboratory-scale synthesis and industrial-scale production. For nano-bioremediation to become an innovation on a commercial scale, researchers must continue to promote it, extensive research on several nanoparticle-related topics is required prior to their commercialization and the government must provide funds for inexpensive and environmentally friendly production.

## Data Availability

The authors declare that the data supporting the findings of this study are available within the paper. Should any raw data files be needed in another format, they are available from the corresponding author upon reasonable request.
